# Supplementary material for: Full genome viral sequences inform patterns of SARS-CoV-2 spread into and within Israel
Source: Nat Commun. 2020 Nov 2;11:5518. doi: 10.1038/s41467-020-19248-0 (PMC7606475; doi:10.1038/s41467-020-19248-0)
Supplement: Supplementary file 1 — Supplementary Information [file 41467_2020_19248_MOESM1_ESM.pdf]

# Full genome viral sequences inform patterns of SARS-CoV-2 spread into and within Israel

Danielle Miller*, Michael A. Martin*, Noam Harel*, Omer Tirosh*, Talia Kustin*, Moran Meir, Nadav Sorek, Shiraz Gefen-Halevi, Sharon Amit, Olesya Vorontsov, Avraham Shaag, Dana Wolf, Avi Peretz, Yonat Shemer-Avni, Diana Roif-Kaminsky, Naama M. Kopelman, Amit Huppert, Katia Koelle, Adi Stern

## Supplementary Information

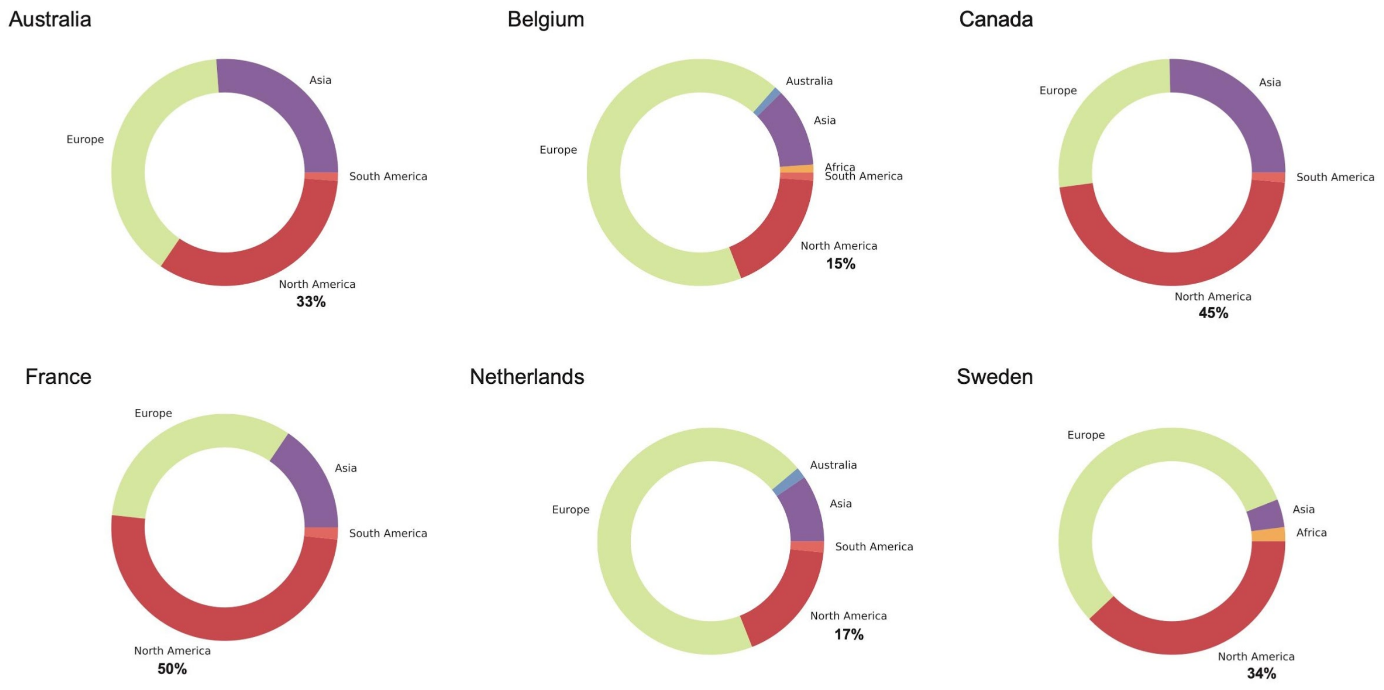


**Supplementary Fig. 1. Inferred regions responsible for clade introductions into six focal countries.** Each of the six countries shown had > 100 sequences available for analysis. Sectors in each pie chart show the proportion of clade introductions that were inferred to originate from the labeled regions. North America is highlighted in bold, and includes almost exclusively sequences from the U.S. For each country, we excluded local transmission (e.g., the Europe sector in the Netherlands excluded the Netherlands).

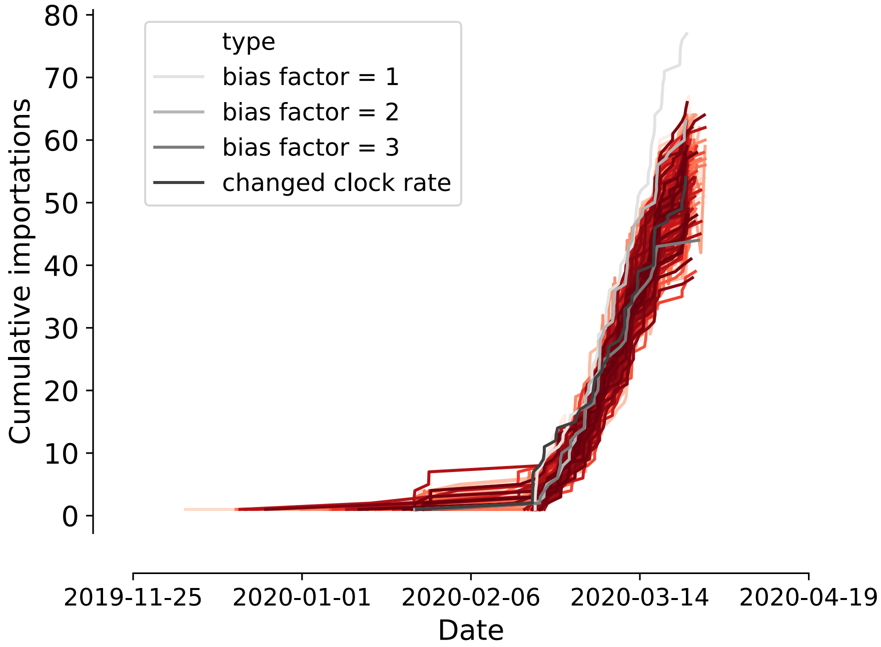


**Supplementary Fig. 2. Cumulative number of importations throughout time.** Cumulative sum of importation events into Israel for 1,000 bootstrap replicates are presented in red spectra. Changes in the sampling correction bias and clock rate are presented in greyscale. Bias factor of 1 means no sampling correction to the GTR matrix of the TreeTime model.

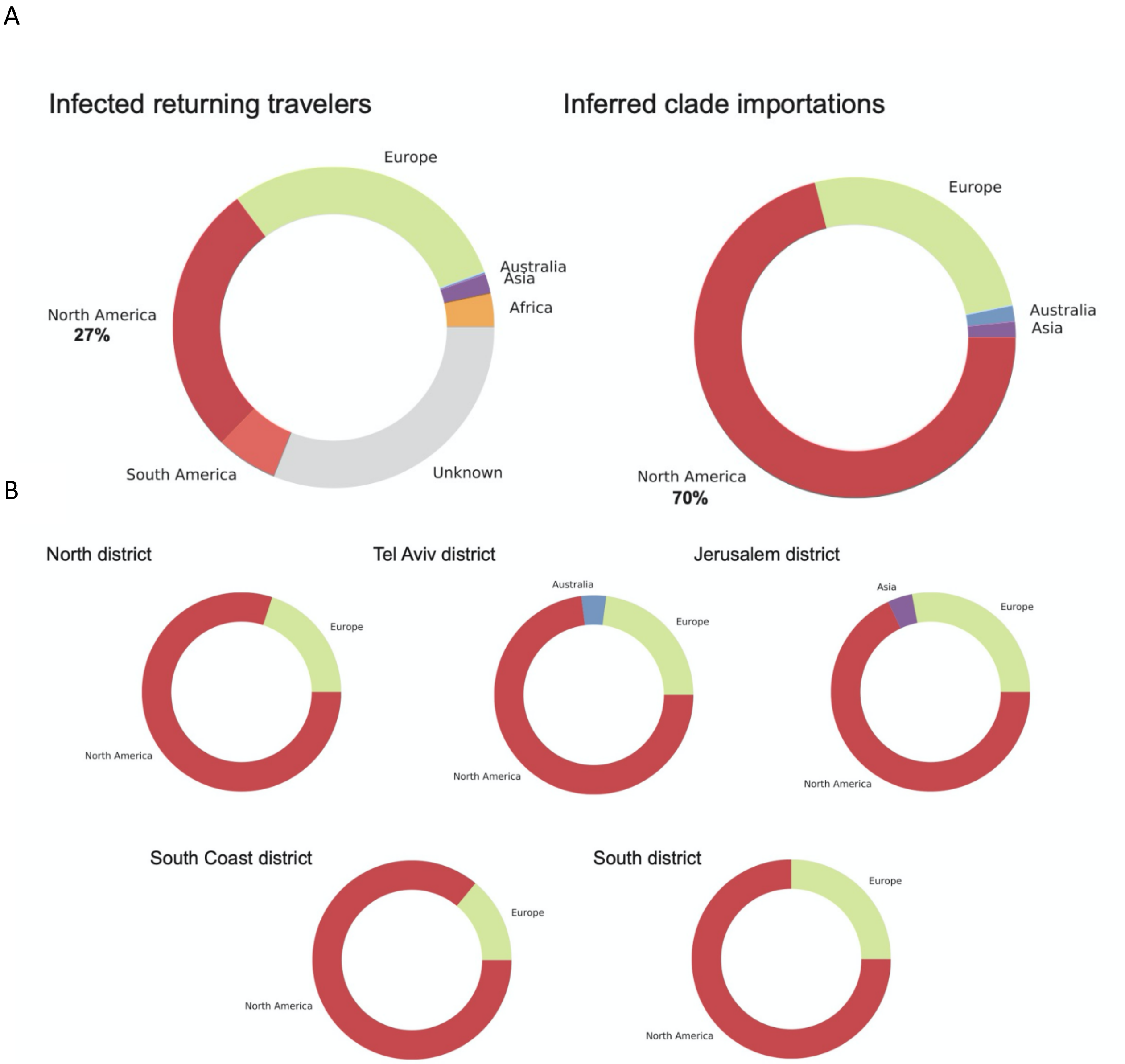


**Supplementary Fig. 3. Distribution of importations into Israel.** (A) Comparison of viral importations into Israel according to epidemiological data (the number of infected travelers reported) versus viral genetic data (inferred clade introductions). (B) Inferred proportion of clade introductions, by district within Israel. As in Figure S1, local transmissions were excluded from the analysis.

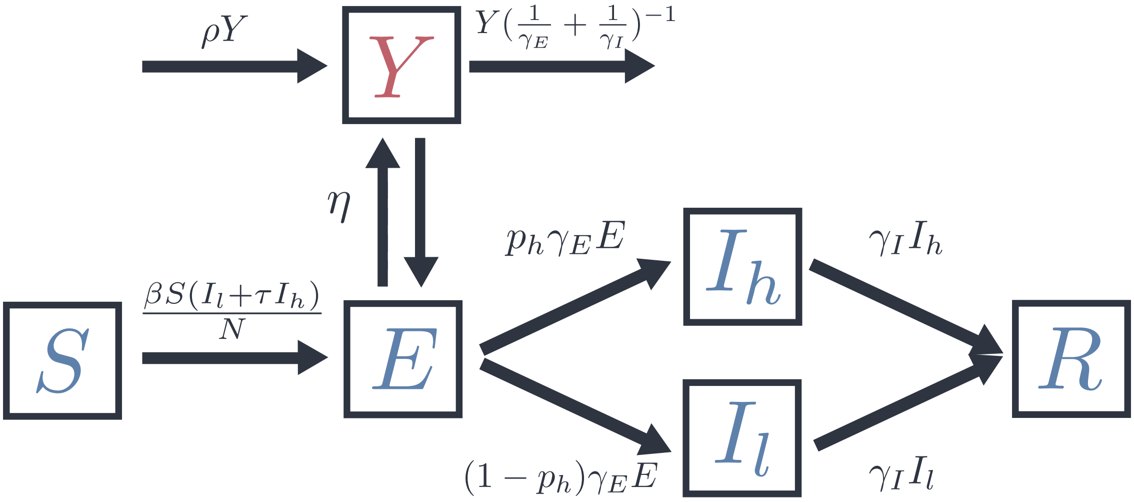


**Supplementary Fig. 4. Compartmental model used in the phylodynamic analysis.** Compartments shown in blue ($S, E, I_h, I_l, R$) represent classes of individuals within Israel. Compartment shown in red ($Y$) represents a single class of infected individuals outside of Israel. The transmission term within Israel is given by $\frac{\beta S(I_l+\tau I_h)}{N}$ where $\tau$ is a factor by which highly infectious individuals ($I_h$) are more infectious than those with lower infectiousness ( $I_l$). The parameter $p_h$ is the probability that an individual transition to the highly infectious ($I_h$) class. The parameter $\tau$ is given by $(\frac{1-p_h}{p_h})/(\frac{1}{0.8}-1)$, such that individuals in $I_h$ are responsible for 80% of secondary infections. Infected individuals remain exposed for $\gamma_E^{-1} =$ 3 days and remain infectious for $\gamma_I^{-1} =$ 5.5 days. Exposed individuals migrate into and out of Israel at an overall rate of $\eta$, which we consider can change over time (discussed in more detail elsewhere in the text). Infected individuals in the exogenous reservoir ($Y$) reproduce at a per capita rate of $\rho$ and individuals remain in this reservoir for and average of 8.5 days.

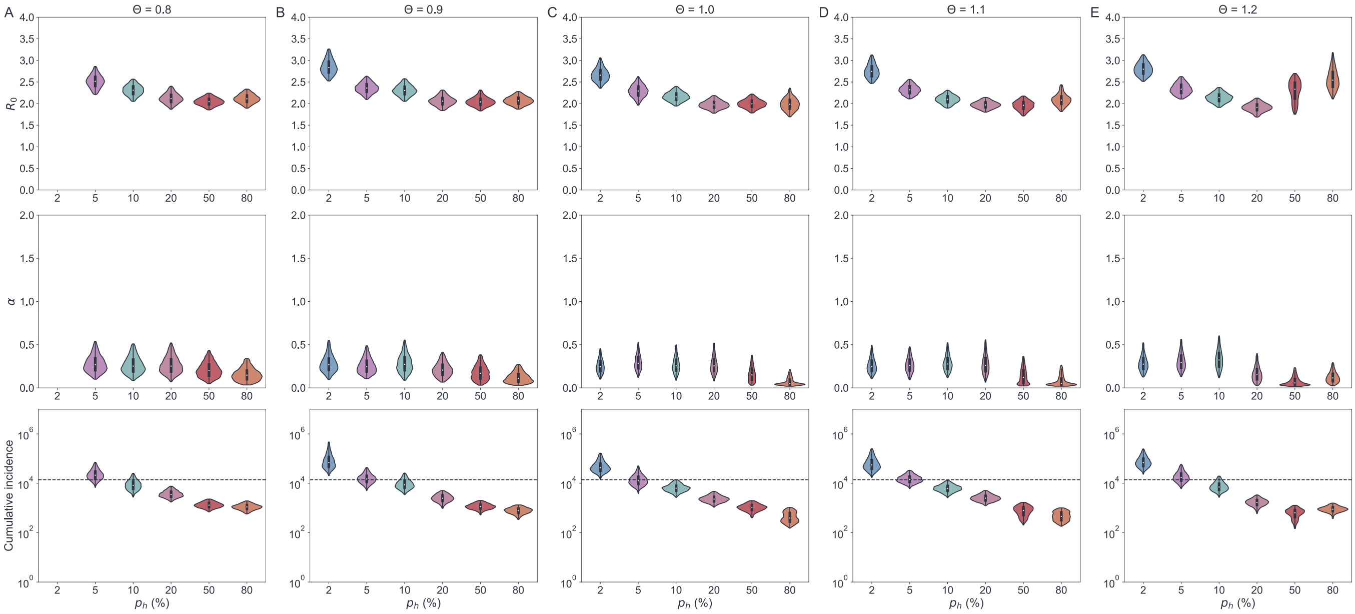


**Supplementary Fig. 5. Sensitivity of phylodynamic analyses to the overall magnitude of the importation/exportation rate $\eta$.** The parameter $\theta$ scales the importation/exportation rate $\eta(t)$ (see Methods). Estimated values of $R_0$, $\alpha$, and cumulative incidence using the migration rate form based on phylogenetically-informed timing of clade introductions. Columns shown correspond to (A) $\theta$ = 0.8, (B) $\theta$ = 0.9, (C) $\theta$ = 1.0 (as shown in Figure 5), (D) $\theta$ = 1.1, (E) $\theta$ = 1.2. Horizontal dotted lines at N = 13,942 in the bottom row show the cumulative number of reported cases on April 22, 2020 as given by the ECDC. Only values which fall within the 95% highest posterior density (HPD) from the main MCMC chain are shown (total of 4,751 data points). Violin plots show the kernel density estimation of the underlying distribution. Median value is denoted by a white dot and the black bar in the center of the violin defines the interquartile range. The upper/lower adjacent values (the black line stretched from the bar) is defined as 1.5 times the width of the interquartile range past the first and third quartiles respectively. Density is only plotted over the range of observed values. Results for $p_h = 0.02,\ \theta = 0.8$ are excluded due to poor mixing of the MCMC chain for this set of parameters.

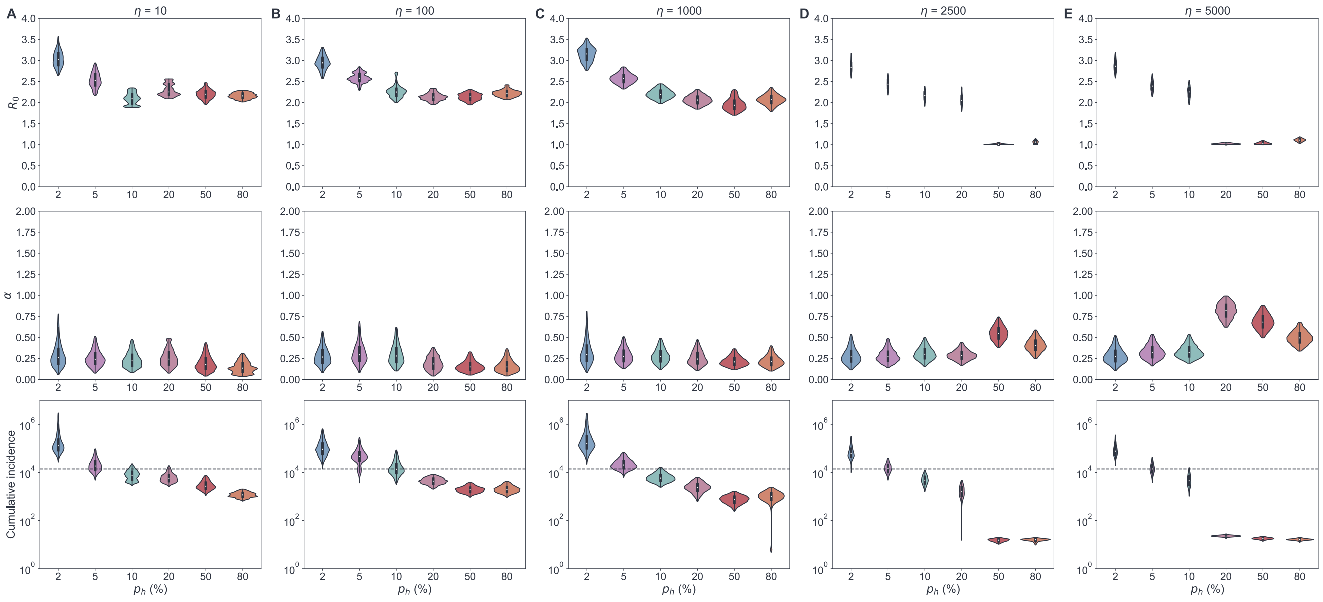


**Supplementary Fig. 6. Sensitivity of phylodynamic analyses to the overall magnitude of the importation/exportation rate $\eta$, when $\eta$ is assumed to be constant.** Columns shown correspond to (A) $\eta$ = 10 (B) $\eta$ = 100, (C) $\eta$ = 1000, (D) $\eta$ = 2500, (E) $\eta$ = 5000 . Horizontal dotted lines at N = 13,942 in the bottom row show the cumulative number of reported cases on April 22, 2020 as given by the ECDC. Only values which fall within the 95% highest posterior density (HPD) from the main MCMC chain are shown (total of 4,751 data points). Violin plots show the kernel density estimation of the underlying distribution. Median value is denoted by a white dot and the black bar in the center of the violin defines the interquartile range. The upper/lower adjacent values (the black line stretched from the bar) is defined as 1.5 times the width of the interquartile range past the first and third quartiles respectively. Density is only plotted over the range of observed values.

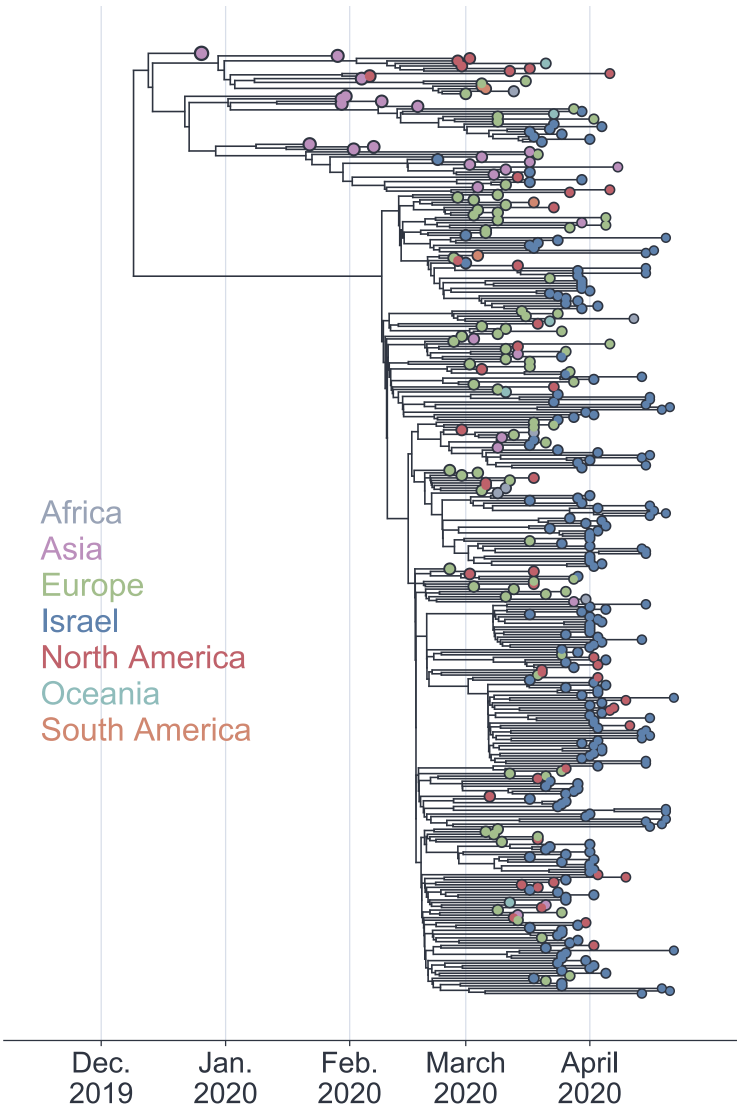


**Supplementary Fig. 7. Time-aligned maximum clade credibility phylogeny.** Phylogeny was estimated using both genetic and epidemiological parameters under an *SEIR* model implemented in BEAST2 and PhyDyn, under $p_h$ = 5% and $\theta = 1.0$. Tips are colored by sampling location with Israel tips shown in blue.

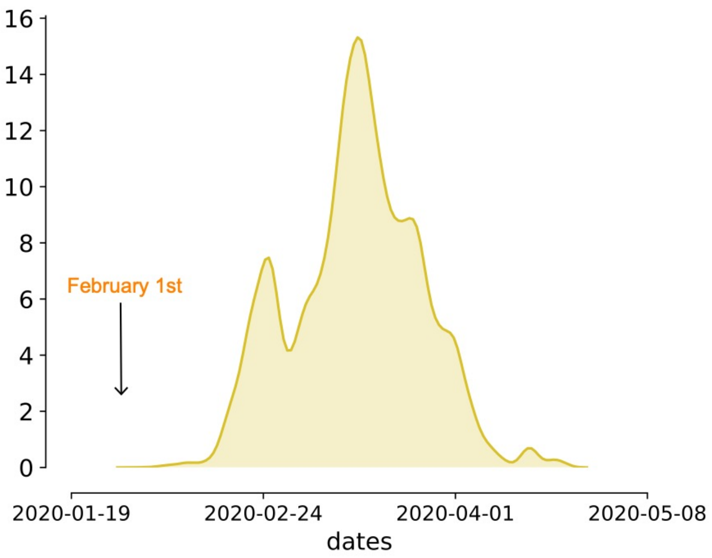


**Supplementary Fig. 8. Distribution of importation dates into Israel.** Density plot of all importation dates inferred from the phylogeny. Earliest importation is around February 1st (marked with an arrow). The highest peak of the distribution is around March 19th, ten days after the official date of airport closure (March 9th).

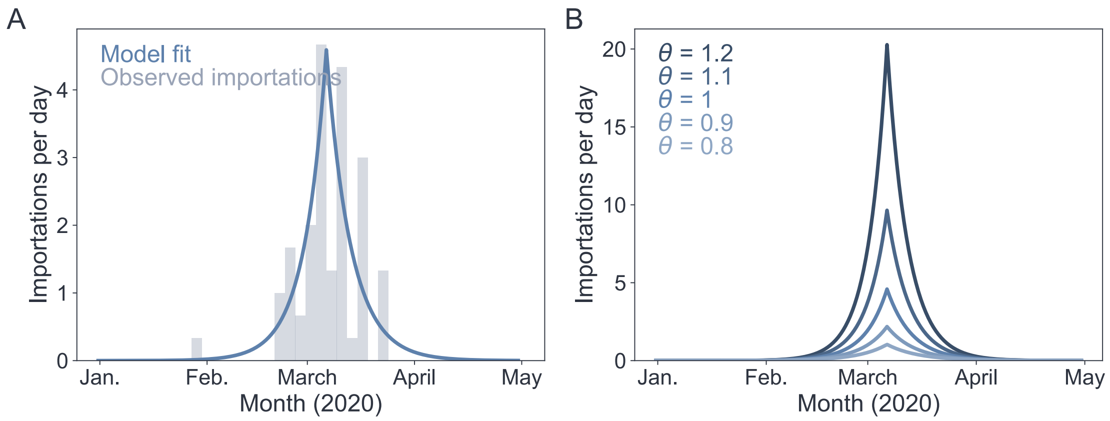


**Supplementary Fig. 9. Distribution of importation dates into Israel assumed in our phylodynamic analysis.** (A) Distribution of importation dates into Israel estimated using ancestral state reconstruction of a global phylogeny. Dates were grouped into non-overlapping three day windows and a piecewise exponential function fit to the data. (B) The growth and decay rates of the inferred importation curve were scaled by a range of $\theta$ values (0.8, 0.9, 1.0, 1.1, 1.2).

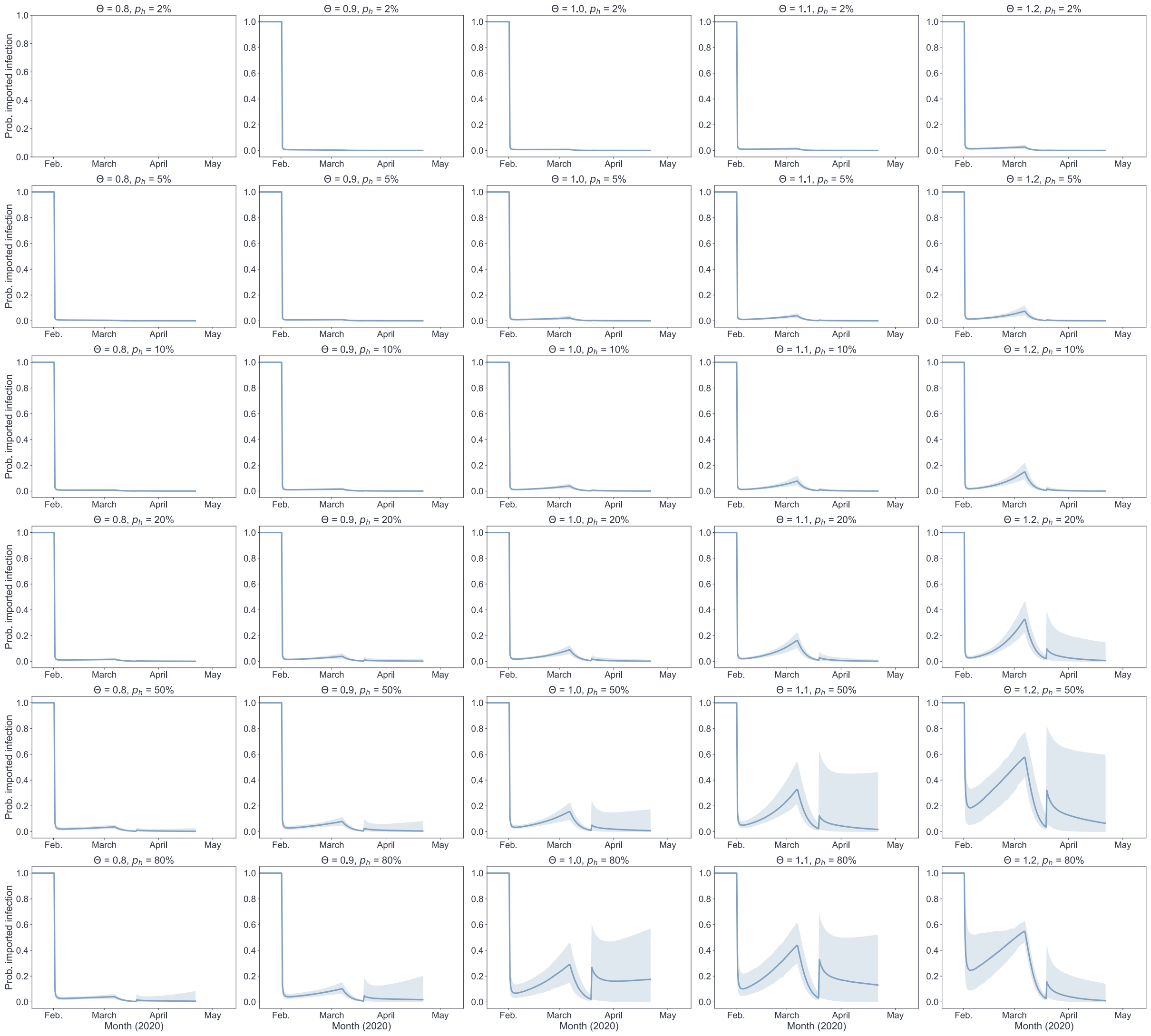


**Supplementary Fig. 10. Probabilities of a new infection being due to importation rather than local infection under different magnitudes of the importation rate, assuming a functional form of $\eta$ based on inferred timing of clade importations.** Estimated state probabilities are shown across a range of $\theta$ (columns) and $p_h$ (rows) values. Line represents the median estimate of the 95% HPD and shaded regions represent the limits of the 95% HPD. Results for $p_h = 0.02,\ \theta = 0.8$ are excluded due to poor mixing of the MCMC chain.

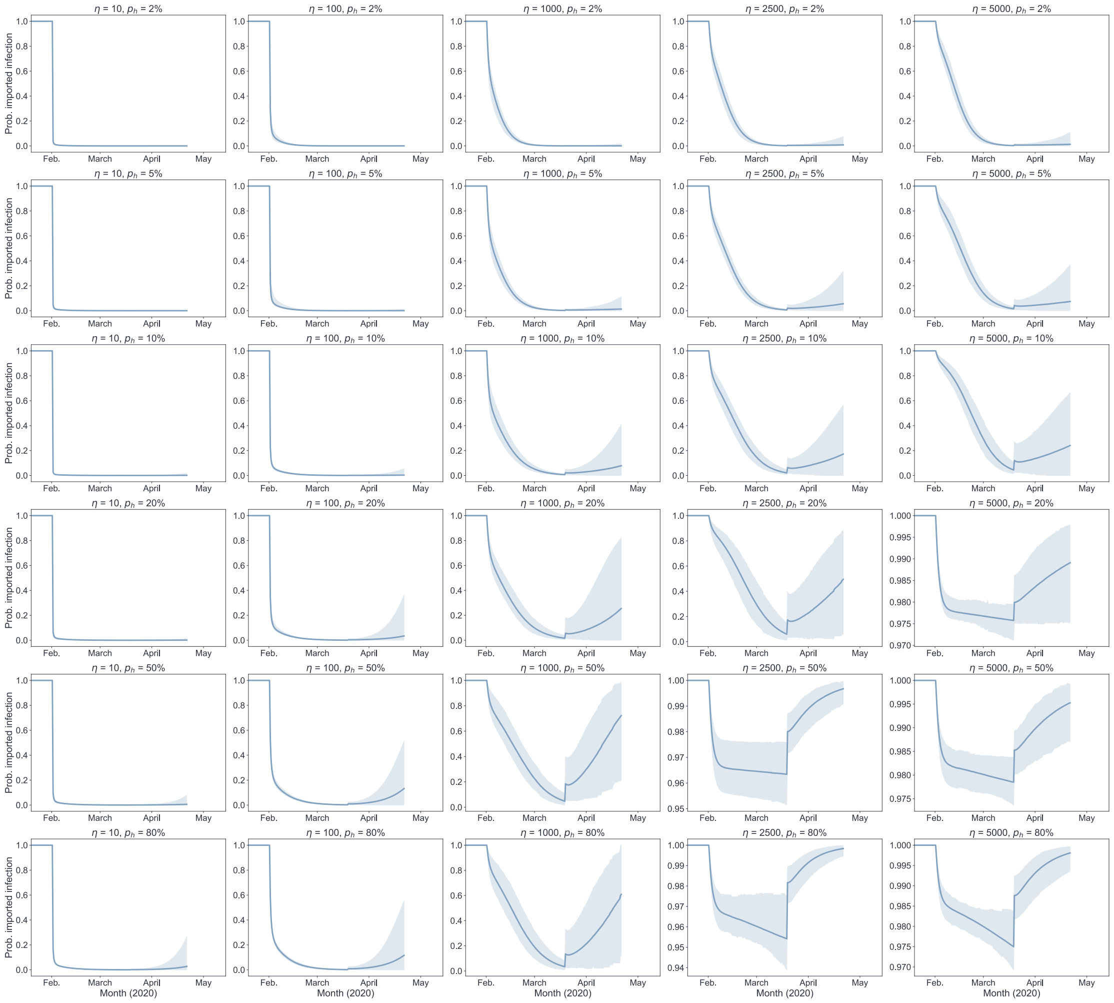


**Supplementary Fig. 11. Probabilities of a new infection being due to importation rather than local infection under different magnitudes of the importation rate, assuming a constant $\eta$.** Estimated state probabilities are shown across a range of $\eta$ values (columns) and $p_h$ values (rows). Line represents the median estimate of the 95% HPD and shaded regions represent the limits of the 95% HPD.

**Supplementary Table 1: Global samples belonging to the S2430R clade that contain short unique deletions.**

A sample was defined as belonging to the S2340R clade if it bore all 6 mutations that define the S2340R clade ('A20755C', 'A23403G', 'C1059T', 'C14408T', 'C3037T', 'G25563T'). The coordinates of the deletions are specified (coordinates based on the Wuhan ancestral sequences, see Methods), as is the country of origin of each sequence.

| EPI_ID | deletion start | deletion end | deletion length | country |
|---|---|---|---|---|
| EPI_ISL_418202 | 26531 | 26531 | 1 | USA |
| EPI_ISL_428377 | 510 | 518 | 9 | USA |
| EPI_ISL_447362 | 3881 | 3898 | 18 | Israel |
| EPI_ISL_447363 | 3881 | 3898 | 18 | Israel |
| EPI_ISL_447377 | 28254 | 28254 | 1 | Israel |
| EPI_ISL_447426 | 27387 | 27396 | 10 | Israel |
| EPI_ISL_450084 | 23555 | 23582 | 28 | USA |
| EPI_ISL_460096 | 29555 | 29602 | 48 | Russia |

**Supplementary Table 2. Details of samples sequenced.**

| Sample ID | Ct E | Ct RdRp | Ct N | Ct average | Sex | Age | Date | Genome coverage | Geog. location of sample |
|---|---|---|---|---|---|---|---|---|---|
| 1639953 | 28.5 | 30.5 | 32 | 30.18 | M | 10-19 | 26/3/20 | 0.996 | North |
| 1639996 | 23.1 | 24.4 | 26 | 24.45 | M | 20-29 | 26/3/20 | 0.996 | North |
| 2020038 | 22.5 | | | 22.46 | F | 50-59 | 17/3/20 | 0.994 | Tel Aviv |
| 2020051 | 30.9 | | | 30.87 | M | 40-49 | 17/3/20 | 0.976 | Tel Aviv |
| 2020063 | 28.4 | | | 28.39 | F | 20-29 | 17/3/20 | 0.985 | Jerusalem |
| 2020068 | 24.8 | | | 24.78 | F | 20-29 | 17/3/20 | 0.986 | Jerusalem |
| 2020069 | 28.2 | | | 28.24 | F | 30-39 | 17/3/20 | 0.987 | Jerusalem |
| 2020078 | 21.5 | | | 21.5 | M | 50-59 | 17/3/20 | 0.989 | Jerusalem |
| 2020084 | 24.9 | | | 24.87 | M | 60-69 | 17/3/20 | 0.994 | Jerusalem |
| 2020087 | 21.5 | | | 21.48 | M | 20-29 | 17/3/20 | 0.996 | Jerusalem |
| 2023920 | 18.6 | | | 18.56 | M | 40-49 | 17/3/20 | 0.996 | Jerusalem |
| 2023922 | 28 | | | 28 | F | 20-29 | 17/3/20 | 0.979 | Jerusalem |
| 2046129 | 23.1 | 24.2 | 27 | 24.88 | M | 20-29 | 31/3/20 | 0.996 | North |
| 2046171 | 29.6 | 30.8 | 33 | 31.24 | F | 50-59 | 31/3/20 | 0.954 | North |
| 2046434 | 19.5 | 20.6 | 24 | 21.28 | F | 50-59 | 1/4/20 | 0.996 | North |
| 2046548 | 28.5 | 31.1 | 34 | 31.01 | M | 40-49 | 1/4/20 | 0.991 | North |
| 2046614 | 24.4 | 26.2 | 28 | 26.24 | F | 30-39 | 2/4/20 | 0.996 | North |
| 2046616 | 20.5 | 22.3 | 25 | 22.47 | F | 70-79 | 2/4/20 | 0.996 | North |
| 2046815 | 13.8 | 16.5 | 18 | 16.08 | F | 80-89 | 4/4/20 | 0.994 | North |
| 2047004 | 23 | 24.5 | 27 | 24.81 | M | 30-39 | 27/3/20 | 0.996 | North |
| 2047011 | 28.5 | 30.2 | 32 | 30.24 | M | 20-29 | 26/3/20 | 0.952 | North |
| 2047016 | 25.7 | 26.7 | 29 | 26.99 | F | 0-9 | 26/3/20 | 0.996 | North |
| 2047145 | 10.3 | 11.8 | 15 | 12.38 | F | 30-39 | 26/3/20 | 0.996 | North |
| 2047188 | 13.6 | 15.3 | 18 | 15.48 | M | 20-29 | 26/3/20 | 0.996 | North |
| 2047189 | 9.75 | 11.7 | 14 | 11.87 | M | 20-29 | 26/3/20 | 0.996 | North |
| 2047364 | 20.8 | 22.8 | 25 | 22.68 | F | 60-69 | 28/3/20 | 0.996 | North |
| 2047392 | 27.6 | 29.9 | 31 | 29.42 | F | 30-39 | 29/3/20 | 0.966 | North |
| 2047418 | 24 | 25.4 | 28 | 25.67 | M | 0-9 | 29/3/20 | 0.996 | North |
| 2047567 | 26.8 | 28.7 | 31 | 28.97 | F | 30-39 | 29/3/20 | 0.978 | North |
| 2047586 | 22.8 | 24.1 | 27 | 24.71 | F | 20-29 | 29/3/20 | 0.996 | North |
| 2047604 | 22.7 | 24 | 26 | 24.32 | M | 50-59 | 29/3/20 | 0.996 | North |
| 2047738 | 20 | 22.1 | 25 | 22.21 | M | 10-19 | 30/3/20 | 0.996 | North |
| 2047749 | 19.3 | 20.7 | 23 | 21.03 | F | 40-49 | 30/3/20 | 0.996 | North |
| 2047772 | 23.8 | 25.7 | 26 | 25.3 | F | 30-39 | 30/3/20 | 0.996 | North |
| 2047883 | 21.1 | 22.9 | 25 | 22.87 | M | 60-69 | 31/3/20 | 0.996 | North |
| 2047927 | 32 | 33.7 | 36 | 33.74 | F | 60-69 | 31/3/20 | 0.918 | North |
| 2086001 | 28.4 | | | 28.4 | F | 10-19 | 1/4/20 | 0.988 | Jerusalem |
| 2086004 | 24.6 | | | 24.63 | F | 20-29 | 1/4/20 | 0.993 | Jerusalem |
| 2086008 | 20.2 | | | 20.24 | M | 90+ | 1/4/20 | 0.996 | Jerusalem |
| 2086012 | 24.4 | | | 24.39 | M | 20-29 | 1/4/20 | 0.988 | Jerusalem |
| 2086022 | 27.9 | | | 27.88 | M | 20-29 | 1/4/20 | 0.986 | Jerusalem |
| 2086033 | 28.8 | | | 28.79 | F | 60-69 | 1/4/20 | 0.991 | Jerusalem |
| 2086034 | 30 | | | 30.01 | F | 20-29 | 1/4/20 | 0.981 | Jerusalem |

| | | | | | | | | | |
|---|---|---|---|---|---|---|---|---|---|
| 2086045 | 20 | | | 19.99 | M | 50-59 | 1/4/20 | 0.996 | Jerusalem |
| 2089366 | 20.4 | | | 20.36 | M | 20-29 | 1/4/20 | 0.996 | Jerusalem |
| 2089368 | 25.6 | | | 25.62 | M | 60-69 | 1/4/20 | 0.985 | Jerusalem |
| 2089375 | 27.5 | | | 27.54 | M | 10-19 | 1/4/20 | 0.992 | Jerusalem |
| 2089380 | 30.1 | | | 30.14 | F | 60-69 | 1/4/20 | 0.979 | Jerusalem |
| 2089383 | 29.8 | | | 29.79 | M | 30-39 | 1/4/20 | 0.989 | Jerusalem |
| 2089697 | 28.3 | | | 28.27 | F | 20-29 | 1/4/20 | 0.989 | Jerusalem |
| 2089698 | 28.6 | | | 28.58 | F | 50-59 | 1/4/20 | 0.984 | Jerusalem |
| 2089712 | 25.5 | | | 25.52 | M | 20-29 | 1/4/20 | 0.989 | Jerusalem |
| 2089718 | 20.6 | | | 20.6 | F | 30-39 | 1/4/20 | 0.996 | Jerusalem |
| 2089723 | 28.6 | | | 28.55 | M | 30-39 | 1/4/20 | 0.992 | Jerusalem |
| 2089812 | 23.3 | | | 23.29 | F | 20-29 | 1/4/20 | 0.995 | Jerusalem |
| 2089839 | 25.5 | | | 25.5 | M | 60-69 | 1/4/20 | 0.989 | Jerusalem |
| 2089852 | 25.5 | | | 25.53 | F | 60-69 | 1/4/20 | 0.989 | Jerusalem |
| 2089861 | 18.3 | | | 18.31 | M | 70-79 | 1/4/20 | 0.996 | Jerusalem |
| 2089863 | 18.8 | | | 18.8 | M | 30-39 | 1/4/20 | 0.996 | Jerusalem |
| 2089866 | 20.7 | | | 20.7 | M | 30-39 | 1/4/20 | 0.996 | Jerusalem |
| 2099018 | 30.3 | | | 30.34 | F | 60-69 | 2/4/20 | 0.980 | Jerusalem |
| 2099019 | 27.5 | | | 27.48 | M | 20-29 | 2/4/20 | 0.992 | Jerusalem |
| 2099159 | 27.3 | | | 27.3 | F | 10-19 | 2/4/20 | 0.988 | Jerusalem |
| 2099251 | 23 | | | 22.98 | F | 20-29 | 2/4/20 | 0.996 | Jerusalem |
| 2099416 | 29 | | | 28.98 | M | 20-29 | 2/4/20 | 0.983 | Jerusalem |
| 2099421 | 20.5 | | | 20.45 | M | 50-59 | 2/4/20 | 0.996 | Jerusalem |
| 2107132 | 29.9 | | | 29.91 | F | 50-59 | 14/4/20 | 0.996 | Jerusalem |
| 2107137 | 19.6 | | | 19.6 | F | 10-19 | 14/4/20 | 0.996 | Jerusalem |
| 2107681 | 22.3 | | | 22.3 | F | 30-39 | 14/4/20 | 0.996 | Jerusalem |
| 2113155 | 25.6 | | | 25.55 | M | 0-9 | 15/4/20 | 0.996 | Jerusalem |
| 2113161 | 25.9 | | | 25.92 | M | 70-79 | 15/4/20 | 0.996 | Jerusalem |
| 2113173 | 29.8 | | | 29.78 | M | 10-19 | 15/4/20 | 0.996 | Jerusalem |
| 2113174 | 26.4 | | | 26.42 | M | 70-79 | 15/4/20 | 0.996 | Tel Aviv |
| 2113178 | 26.6 | | | 26.62 | M | 20-29 | 15/4/20 | 0.996 | Jerusalem |
| 2113255 | 29.1 | | | 29.14 | F | 60-69 | 14/4/20 | 0.994 | Jerusalem |
| 2113256 | 29.7 | | | 29.65 | F | 10-19 | 14/4/20 | 0.996 | Jerusalem |
| 2113601 | 25.1 | | | 25.07 | M | 0-9 | 15/4/20 | 0.996 | Jerusalem |
| 2113603 | 21.7 | | | 21.72 | M | 70-79 | 15/4/20 | 0.996 | Jerusalem |
| 2113678 | 29.8 | | | 29.78 | M | 0-9 | 15/4/20 | 0.996 | Tel Aviv |
| 2115701 | 26 | | | 25.96 | F | 10-19 | 16/4/20 | 0.996 | Jerusalem |
| 2115964 | 28.4 | | | 28.35 | M | 10-19 | 16/4/20 | 0.996 | Tel Aviv |
| 2115968 | 26.8 | | | 26.83 | F | 20-29 | 16/4/20 | 0.996 | Tel Aviv |
| 2115976 | 28.2 | | | 28.17 | M | 60-69 | 16/4/20 | 0.985 | Tel Aviv |
| 2115980 | 24.5 | | | 24.49 | F | 20-29 | 16/4/20 | 0.912 | North |
| 2115990 | 30.2 | | | 30.15 | F | 50-59 | 16/4/20 | 0.996 | Jerusalem |
| 2116859 | 29.6 | | | 29.6 | F | 10-19 | 20/4/20 | 0.953 | Jerusalem |
| 2123853 | 28.1 | | | 28.05 | M | 70-79 | 20/4/20 | 0.996 | Jerusalem |
| 2123863 | 27.5 | | | 27.47 | M | 80-89 | 20/4/20 | 0.996 | Jerusalem |
| 13075703 | 24 | 25.6 | 28 | 25.98 | F | 20-29 | 29/3/20 | 0.996 | Tel Aviv |
| 13075719 | 27.7 | 28.9 | 31 | 29.16 | F | 20-29 | 29/3/20 | 0.996 | Tel Aviv |
| 13075735 | 26.8 | 28.4 | 30 | 28.47 | M | 40-49 | 30/3/20 | 0.996 | Tel Aviv |
| 13075782 | 29.1 | 30.5 | 33 | 30.85 | F | 40-49 | 30/3/20 | 0.992 | Tel Aviv |

| | | | | | | | | | |
|---|---|---|---|---|---|---|---|---|---|
| 13075788 | 27.8 | 29.3 | 31 | 29.4 | F | 40-49 | 30/3/20 | 0.996 | Tel Aviv |
| 13075790 | 27.2 | 28.8 | 30 | 28.7 | M | 50-59 | 30/3/20 | 0.996 | Tel Aviv |
| 13075832 | 17.5 | 18.6 | 20 | 18.7 | M | 30-39 | 30/3/20 | 0.996 | Tel Aviv |
| 13075879 | 18.6 | 20 | 22 | 20.27 | M | 50-59 | 30/3/20 | 0.993 | Tel Aviv |
| 13075882 | 18.6 | 20.4 | 21 | 20.1 | F | 20-29 | 30/3/20 | 0.996 | Tel Aviv |
| 13075914 | 17.1 | 18.5 | 20 | 18.39 | F | 50-59 | 30/3/20 | 0.996 | Tel Aviv |
| 13077377 | 27 | 29 | 29 | 28.33 | M | 40-49 | 2/4/20 | 0.996 | Tel Aviv |
| 13077383 | 26 | 26 | 28 | 26.67 | F | 40-49 | 2/4/20 | 0.984 | Jerusalem |
| 13077413 | 24 | 25 | 26 | 25 | M | 70-79 | 3/4/20 | 0.996 | Tel Aviv |
| 13077494 | 28 | 29 | 31 | 29.33 | M | 20-29 | 2/4/20 | 0.856 | South Coast |
| 13077497 | 21 | 22 | 24 | 22.33 | F | 60-69 | 2/4/20 | 0.996 | South Coast |
| 13077498 | 17 | 18 | 21 | 18.67 | M | 20-29 | 3/4/20 | 0.996 | South Coast |
| 13077510 | 21 | 22 | 25 | 22.67 | M | 50-59 | 3/4/20 | 0.993 | Jerusalem |
| 13077511 | 25 | 26 | 28 | 26.33 | M | 20-29 | 3/4/20 | 0.986 | Jerusalem |
| 13077558 | 19 | 20 | 22 | 20.33 | F | 30-39 | 3/4/20 | 0.996 | Tel Aviv |
| 13077560 | 27 | 28 | 30 | 28.33 | F | 80-89 | 3/4/20 | 0.974 | Tel Aviv |
| 13077562 | 15 | 17 | 19 | 17 | F | 50-59 | 3/4/20 | 0.988 | Tel Aviv |
| 13077564 | 22 | 23 | 25 | 23.33 | F | 50-59 | 3/4/20 | 0.992 | Tel Aviv |
| 13077711 | 27 | 28 | 30 | 28.33 | F | 30-39 | 3/4/20 | 0.995 | Tel Aviv |
| 13077723 | 22 | 23 | 25 | 23.33 | M | ? | 3/4/20 | 0.989 | Tel Aviv |
| 13077726 | 20 | 20 | 22 | 20.67 | F | 60-69 | 3/4/20 | 0.996 | Tel Aviv |
| 13077803 | 28 | 29 | 32 | 29.67 | F | 30-39 | 4/4/20 | 0.981 | South Coast |
| 13077823 | 15 | 17 | 19 | 17 | F | 10-19 | 4/4/20 | 0.996 | South Coast |
| 13077840 | 23 | 25 | 27 | 25 | M | 20-29 | 4/4/20 | 0.991 | Jerusalem |
| 13077846 | 19 | 20 | 23 | 20.67 | F | 0-9 | 4/4/20 | 0.996 | Tel Aviv |
| 13077847 | 24 | 26 | 27 | 25.67 | M | 10-19 | 4/4/20 | 0.986 | Tel Aviv |
| 13077875 | 18 | 19 | 21 | 19.33 | F | 40-49 | 4/4/20 | 0.996 | Tel Aviv |
| 13077882 | 24 | 26 | 27 | 25.67 | M | 40-49 | 4/4/20 | 0.987 | Jerusalem |
| 51137031 | | | | 18.58 | M | 30-39 | 1/3/20 | 0.996 | Tel Aviv |
| 51137844 | | | | 27.6 | F | 30-39 | 17/3/20 | 0.983 | South |
| 51140028 | | | | 20.56 | F | 50-59 | 17/3/20 | 0.996 | North |
| 51140068 | | | | 22.8 | M | 30-39 | 18/3/20 | 0.988 | South |
| 51140271 | | | | 28.16 | F | 40-49 | 18/3/20 | 0.971 | South Coast |
| 51140279 | | | | 25.76 | F | 20-29 | 18/3/20 | 0.976 | South Coast |
| 51140315 | | | | 23.41 | M | 60-69 | 18/3/20 | 0.996 | South |
| 51140539 | | | | 19.4 | F | 50-59 | 19/3/20 | 0.996 | South |
| 51140836 | | | | 24.5 | M | 30-39 | 20/3/20 | 0.986 | South |
| 51141014 | | | | 23.08 | F | 90+ | 22/3/20 | 0.996 | South |
| 51141121 | | | | 29.05 | M | 70-79 | 22/3/20 | 0.989 | South |
| 51141225 | | | | 18.58 | F | 40-49 | 22/3/20 | 0.996 | South |
| 51144342 | | | | 25.59 | M | 90+ | 30/3/20 | 0.976 | South |
| 51145198 | | | | 25.49 | M | 80-89 | 1/4/20 | 0.989 | South |
| 51145482 | | | | 27.92 | F | 40-49 | 5/4/20 | 0.992 | Tel Aviv |
| 51146355 | | | | 29.24 | F | 80-89 | 4/4/20 | 0.989 | South |
| 51146500 | | | | 21.79 | M | 40-49 | 5/4/20 | 0.989 | Tel Aviv |
| 51146503 | | | | 23.75 | F | 30-39 | 5/4/20 | 0.993 | Jerusalem |
| 51146669 | | | | 24.41 | M | 10-19 | 5/4/20 | 0.994 | Tel Aviv |
| 51146683 | | | | 24.8 | M | 10-19 | 5/4/20 | 0.965 | Tel Aviv |
| 130710062 | 28 | 28 | 28 | 28 | M | 40-49 | 14/4/20 | 0.991 | Tel Aviv |

| | | | | | | | | | |
|---|---|---|---|---|---|---|---|---|---|
| 130710067 | 23 | 24 | 24 | 23.67 | M | 60-69 | 14/4/20 | 0.996 | Tel Aviv |
| 130710097 | 29 | 31 | 32 | 30.67 | M | 70-79 | 15/4/20 | 0.996 | Tel Aviv |
| 130710099 | 33 | 33 | 34 | 33.33 | M | 60-69 | 15/4/20 | 0.979 | Tel Aviv |
| 130710157 | 20 | 21 | 24 | 21.67 | M | 70-79 | 15/4/20 | 0.996 | Tel Aviv |
| 130710159 | 27 | 28 | 29 | 28 | F | 80-89 | 15/4/20 | 0.990 | Tel Aviv |
| 130710211 | 18 | 19 | 20 | 19 | F | 70-79 | 16/4/20 | 0.996 | Tel Aviv |
| 130710217 | 22 | 23 | 26 | 23.67 | M | 50-59 | 16/4/20 | 0.996 | Tel Aviv |
| 130710390 | 28 | 29 | 30 | 29 | F | 30-39 | 17/4/20 | 0.996 | Tel Aviv |
| 130710414 | 20 | 21 | 22 | 21 | M | 20-29 | 17/4/20 | 0.996 | Tel Aviv |
| 130710643 | 31 | 31 | 31 | 31 | M | 40-49 | 19/4/20 | 0.986 | Tel Aviv |
| 130710644 | 29 | 30 | 31 | 30 | F | 80-89 | 19/4/20 | 0.985 | Tel Aviv |
| 130710716 | 30 | 31 | 31 | 30.67 | F | 60-69 | 19/4/20 | 0.989 | Tel Aviv |
| 130711082 | 18.5 | 20 | 22 | 20.17 | F | 60-69 | 20/4/20 | 0.996 | Tel Aviv |
| 130711104 | 27 | 29 | 29 | 28.33 | F | 80-89 | 20/4/20 | 0.993 | Tel Aviv |
| 130711112 | 31 | 32 | 31 | 31.33 | F | 80-89 | 21/4/20 | 0.991 | Tel Aviv |
| 130711116 | 25 | 27 | 25 | 25.67 | F | 60-69 | 21/4/20 | 0.996 | Tel Aviv |
| 130711367 | 29 | 27 | 27 | 27.67 | F | 60-69 | 22/4/20 | 0.996 | North |
| 130711417 | 32 | 31 | 30 | 31 | M | 60-69 | 22/4/20 | 0.974 | Tel Aviv |
| 701002313 | | | | 27 | M | 50-59 | 21/3/20 | 0.996 | South Coast |
| 701002314 | | | | 21 | M | 20-29 | 21/3/20 | 0.996 | South Coast |
| 701002317 | | | | 25 | M | 50-59 | 21/3/20 | 0.996 | South Coast |
| 701002327 | | | | 19 | M | 50-59 | 22/3/20 | 0.996 | South Coast |
| 701002334 | | | | 21 | M | 70-79 | 22/3/20 | 0.996 | South Coast |
| 701002403 | | | | 31 | M | 40-49 | 23/3/20 | 0.996 | South Coast |
| 701002407 | | | | 27 | M | 40-49 | 23/3/20 | 0.959 | South Coast |
| 701002426 | | | | 25 | M | 60-69 | 24/3/20 | 0.996 | South Coast |
| 701002431 | | | | 18 | M | 20-29 | 24/3/20 | 0.996 | South Coast |
| 701002440 | | | | 26 | F | 20-29 | 24/3/20 | 0.996 | South Coast |
| 701002442 | | | | 18 | M | 30-39 | 24/3/20 | 0.996 | South Coast |
| 701002455 | | | | 30 | F | 20-29 | 24/3/20 | 0.992 | South Coast |
| 701002456 | | | | 30 | F | 60-69 | 24/3/20 | 0.980 | South Coast |
| 701002458 | | | | 21 | M | 40-49 | 24/3/20 | 0.996 | South Coast |
| 701002462 | | | | 21 | M | 70-79 | 24/3/20 | 0.996 | South Coast |
| 701002489 | | | | 27 | M | 40-49 | 25/3/20 | 0.996 | South Coast |
| 701002504 | | | | 21 | M | 70-79 | 25/3/20 | 0.996 | South Coast |
| 701002538 | | | | 28 | M | 70-79 | 25/3/20 | 0.996 | South Coast |
| 701002540 | | | | 21 | F | 40-49 | 25/3/20 | 0.996 | South Coast |
| 701002550 | | | | 24 | M | 30-39 | 26/3/20 | 0.996 | South Coast |
| 701002555 | | | | 23 | F | 40-49 | 26/3/20 | 0.996 | South Coast |
| 701002556 | | | | 22 | F | 60-69 | 26/3/20 | 0.996 | South Coast |
| 701002561 | | | | 28 | M | 50-59 | 26/3/20 | 0.996 | South Coast |
| 701002591 | | | | 24 | F | 50-59 | 26/3/20 | 0.996 | South Coast |
| 701002666 | | | | 26 | M | 40-49 | 26/3/20 | 0.996 | South Coast |
| 701002681 | | | | 24 | M | 60-69 | 27/3/20 | 0.996 | South Coast |
| 701002752 | | | | 27 | M | 10-19 | 28/3/20 | 0.987 | South Coast |
| 701002768 | | | | 21 | M | 50-59 | 28/3/20 | 0.996 | South Coast |
| 701002786 | | | | 27 | M | 60-69 | 29/3/20 | 0.996 | South Coast |
| 701002792 | | | | 25 | M | 50-59 | 29/3/20 | 0.995 | South Coast |
| 990059202 | 26.9 | 28.4 | 27 | 27.4 | M | 60-69 | 25/3/20 | 0.996 | South Coast |

| | | | | | | | | | |
|---|---|---|---|---|---|---|---|---|---|
| 990059203 | 26.5 | 28.6 | 27 | 27.2 | F | 80-89 | 25/3/20 | 0.996 | South Coast |
| 990059204 | 28.4 | 30.1 | 28 | 28.97 | F | 30-39 | 25/3/20 | 0.976 | South Coast |
| 990059217 | 29.1 | 31.4 | 32 | 30.9 | M | 60-69 | 25/3/20 | 0.995 | South Coast |
| 990059230 | 26.5 | 29.7 | 29 | 28.5 | F | 0-9 | 25/3/20 | 0.972 | South Coast |
| 990059231 | 24.9 | 28 | 28 | 27 | M | 30-39 | 25/3/20 | 0.996 | South Coast |
| 990059232 | 24.5 | 26.7 | 28 | 26.3 | M | 10-19 | 25/3/20 | 0.996 | South Coast |
| 990059233 | 19.7 | 21.8 | 23 | 21.43 | F | 50-59 | 25/3/20 | 0.996 | South Coast |
| 990059237 | 28.3 | 30.6 | 32 | 30.23 | F | 0-9 | 25/3/20 | 0.989 | South Coast |
| 990059238 | 24.3 | 26.3 | 27 | 25.83 | F | 20-29 | 25/3/20 | 0.996 | South Coast |
| 990059244 | 27.9 | 30.8 | 31 | 29.97 | F | ? | 25/3/20 | 0.938 | South Coast |
| 990059251 | 24.7 | 28 | 28 | 26.9 | F | 50-59 | 25/3/20 | 0.996 | South Coast |
| 990059252 | 24.4 | 26.8 | 28 | 26.27 | F | 20-29 | 25/3/20 | 0.996 | South Coast |
| 990300681 | 22.9 | 26.4 | 27 | 25.37 | F | 60-69 | 30/3/20 | 0.995 | South Coast |
| 990300691 | 29.3 | 31.7 | 32 | 31 | M | 20-29 | 31/3/20 | 0.970 | South Coast |
| 990300724 | 23.7 | 26.6 | 26 | 25.53 | M | 80-89 | 1/4/20 | 0.993 | South Coast |
| 990300860 | 19.3 | 21.9 | 0 | 20.6 | M | 20-29 | 2/4/20 | 0.996 | South Coast |
| 990307712 | 26 | 27.3 | 28 | 26.97 | F | 20-29 | 26/3/20 | 0.996 | South Coast |
| 990333068 | 30.1 | 32.7 | 34 | 32.13 | M | 60-69 | 24/3/20 | 0.963 | South Coast |
| 990333189 | 29.3 | 31.4 | 32 | 30.9 | M | 50-59 | 26/3/20 | 0.950 | South Coast |
| 990333193 | 20.7 | 22.6 | 24 | 22.43 | F | 40-49 | 26/3/20 | 0.996 | South Coast |
| 990333263 | 36.4 | 0 | 0 | 36.4 | M | 30-39 | 31/3/20 | 0.660 | South Coast |
| 990430264 | 29.3 | 31.7 | 33 | 31.17 | F | 40-49 | 29/3/20 | 0.970 | South Coast |
| 990430265 | 29.6 | 32.2 | 33 | 31.7 | F | 30-39 | 29/3/20 | 0.994 | South Coast |

**Supplementary Table 3: Model parameter priors and estimated values (θ = 0.8)**

| | | Posterior (Median (95% HPD)) | | | | | |
|---|---|---|---|---|---|---|---|
| **Parameter** | **Prior** | $p_h = 0.02$ | $p_h = 0.05$ | $p_h = 0.10$ | $p_h = 0.20$ | $p_h = 0.50$ | $p_h = 0.80$ |
| Posterior | | | -46413 (-46465 -46358) | -46425 (-46483 -46357) | -46406 (-46469 -46343) | -46401 (-46462 -46347) | -46421 (-46481 -46360) |
| Tree height | | | 0.377 (0.326 0.443) | 0.384 (0.330 0.455) | 0.367 (0.323 0.434) | 0.356 (0.322 0.413) | 0.358 (0.323 0.426) |
| Clock rate | Uniform(5.0e-4, 2.0e-3) | | 7.37e-04 (6.10e-04 8.83e-04) | 7.36e-04 (6.07e-04 8.87e-04) | 7.81e-04 (6.22e-04 9.26e-04) | 8.09e-04 (6.84e-04 9.24e-04) | 7.68e-04 (6.57e-04 8.87e-04) |
| κ | Lognormal(M=1.0, SD=1.25) | | 3.719 (3.111 4.484) | 3.738 (3.076 4.463) | 3.702 (3.028 4.422) | 3.720 (3.125 4.499) | 3.710 (3.061 4.444) |
| γ | Exponential(M=1.0) | | 3.51e-02 (1.05e-03 8.99e-02) | 3.53e-02 (1.02e-03 8.85e-02) | 3.90e-02 (1.08e-03 9.32e-02) | 3.78e-02 (1.59e-03 8.75e-02) | 3.71e-02 (1.01e-03 8.85e-02) |
| $R_0$ | Lognormal(M=1.5, SD=0.5) | | 2.52 (2.21 2.86) | 2.31 (2.05 2.56) | 2.11 (1.87 2.40) | 2.04 (1.86 2.24) | 2.11 (1.89 2.33) |
| α | Uniform(0.0, 2.0) | | 0.27 (0.10 0.54) | 0.25 (0.09 0.51) | 0.25 (0.07 0.52) | 0.20 (0.05 0.43) | 0.15 (0.03 0.34) |
| $E_{init}$ | Exponential(M=1.0) | | 12.06 (6.42 19.44) | 10.19 (5.80 15.62) | 8.80 (4.59 13.85) | 4.60 (2.52 7.46) | 3.25 (1.90 5.15) |
| $Y_{init}$ | Exponential(M=1.0) | | 6.61e-03 (1.88e-03 1.64e-02) | 6.72e-03 (2.34e-03 1.40e-02) | 3.44e-03 (1.52e-03 1.49e-02) | 2.89e-03 (1.87e-03 4.48e-03) | 3.35e-03 (2.17e-03 6.23e-03) |

**Supplementary Table 4: Model parameter priors and estimated values (θ = 0.9)**

| Parameter | Prior | Posterior (Median (95% HPD)) | | | | | |
|---|---|---|---|---|---|---|---|
| | | $p_h$ = 0.02 | $p_h$ = 0.05 | $p_h$ = 0.10 | $p_h$ = 0.20 | $p_h$ = 0.50 | $p_h$ = 0.80 |
| Posterior | | -46384 (-46439 -46323) | -46395 (-46458 -46334) | -46419 (-46473 -46363) | -46388 (-46454 -46326) | -46396 (-46466 -46335) | -46392 (-46451 -46325) |
| Tree height | | 0.361 (0.323 0.430) | 0.370 (0.323 0.428) | 0.380 (0.325 0.445) | 0.362 (0.323 0.426) | 0.360 (0.323 0.426) | 0.360 (0.322 0.421) |
| Clock rate | Uniform(5.0e-4, 2.0e-3) | 7.74e-04 (6.21e-04 8.97e-04) | 7.84e-04 (6.63e-04 9.06e-04) | 7.37e-04 (6.18e-04 8.63e-04) | 8.13e-04 (6.91e-04 9.53e-04) | 8.03e-04 (6.83e-04 9.24e-04) | 7.98e-04 (6.94e-04 9.16e-04) |
| κ | Lognormal(M=1.0, SD=1.25) | 3.738 (3.104 4.464) | 3.743 (3.148 4.552) | 3.704 (3.057 4.493) | 3.694 (3.093 4.511) | 3.732 (3.040 4.401) | 3.712 (3.090 4.425) |
| γ | Exponential(M=1.0) | 3.55e-02 (1.00e-03 8.73e-02) | 3.80e-02 (1.16e-03 8.87e-02) | 3.83e-02 (1.00e-03 8.83e-02) | 3.55e-02 (1.01e-03 8.91e-02) | 3.97e-02 (1.90e-03 8.98e-02) | 3.84e-02 (1.02e-03 9.09e-02) |
| $R_0$ | Lognormal(M=1.5, SD=0.5) | 2.84 (2.52 3.26) | 2.36 (2.10 2.63) | 2.30 (2.05 2.57) | 2.06 (1.84 2.31) | 2.04 (1.83 2.31) | 2.07 (1.87 2.28) |
| α | Uniform(0.0, 2.0) | 0.27 (0.10 0.55) | 0.24 (0.11 0.49) | 0.27 (0.09 0.55) | 0.21 (0.07 0.41) | 0.17 (0.03 0.38) | 0.11 (0.03 0.27) |
| $E_{init}$ | Exponential(M=1.0) | 12.47 (6.68 19.79) | 15.30 (9.03 23.18) | 10.47 (5.76 16.18) | 8.42 (4.88 12.97) | 4.27 (1.85 7.04) | 2.90 (1.54 4.79) |
| $Y_{init}$ | Exponential(M=1.0) | 5.39e-03 (3.01e-03 1.23e-02) | 4.56e-03 (2.50e-03 8.35e-03) | 6.79e-03 (2.41e-03 1.45e-02) | 3.26e-03 (1.70e-03 7.37e-03) | 3.27e-03 (1.96e-03 5.16e-03) | 3.35e-03 (2.24e-03 4.93e-03) |

**Supplementary Table 5: Model parameter priors and estimated values (θ = 1.0)**

| Parameter | Prior | Posterior (Median (95% HPD)) | | | | | |
|---|---|---|---|---|---|---|---|
| | | $p_h$ = 0.02 | $p_h$ = 0.05 | $p_h$ = 0.10 | $p_h$ = 0.20 | $p_h$ = 0.50 | $p_h$ = 0.80 |
| Posterior | | -46363 (-46426 -46311) | -46370 (-46436 -46310) | -46392 (-46441 -46330) | -46374 (-46436 -46325) | -46396 (-46455 -46339) | -46347 (-46442 -46276) |
| Tree height | | 0.368 (0.323 0.437) | 0.358 (0.323 0.429) | 0.360 (0.322 0.420) | 0.359 (0.322 0.414) | 0.361 (0.323 0.420) | 0.360 (0.323 0.420) |
| Clock rate | Uniform(5.0e-4, 2.0e-3) | 8.01e-04 (6.49e-04 9.37e-04) | 8.24e-04 (6.73e-04 9.97e-04) | 7.87e-04 (6.82e-04 8.98e-04) | 8.41e-04 (7.21e-04 9.55e-04) | 7.98e-04 (6.82e-04 9.03e-04) | 8.35e-04 (7.04e-04 9.62e-04) |
| κ | Lognormal(M=1.0, SD=1.25) | 3.770 (3.041 4.455) | 3.737 (3.055 4.444) | 3.743 (3.099 4.500) | 3.710 (2.982 4.388) | 3.704 (3.087 4.432) | 3.732 (3.067 4.526) |
| γ | Exponential(M=1.0) | 3.76e-02 (1.01e-03 9.23e-02) | 3.81e-02 (1.05e-03 9.01e-02) | 3.79e-02 (1.10e-03 9.37e-02) | 3.91e-02 (1.04e-03 8.88e-02) | 3.89e-02 (1.14e-03 8.76e-02) | 3.56e-02 (1.04e-03 8.58e-02) |
| $R_0$ | Lognormal(M=1.5, SD=0.5) | 2.66 (2.36 3.06) | 2.29 (1.96 2.62) | 2.16 (1.94 2.40) | 1.97 (1.78 2.18) | 1.99 (1.78 2.22) | 1.98 (1.69 2.35) |
| α | Uniform(0.0, 2.0) | 0.25 (0.10 0.45) | 0.29 (0.12 0.53) | 0.26 (0.09 0.50) | 0.26 (0.08 0.52) | 0.15 (0.03 0.38) | 0.05 (0.02 0.21) |
| $E_{init}$ | Exponential(M=1.0) | 15.33 (7.65 24.34) | 15.92 (7.50 26.28) | 13.07 (7.63 19.05) | 9.60 (5.76 13.70) | 4.79 (2.82 7.16) | 2.27 (0.44 3.94) |
| $Y_{init}$ | Exponential(M=1.0) | 5.77e-03 (2.51e-03 1.33e-02) | 4.38e-03 (2.34e-03 9.65e-03) | 4.56e-03 (2.70e-03 8.19e-03) | 3.22e-03 (1.98e-03 5.29e-03) | 3.64e-03 (2.42e-03 5.72e-03) | 3.84e-03 (2.36e-03 5.96e-03) |

**Supplementary Table 6: Model parameter priors and estimated values (θ = 1.1)**

| | | Posterior (Median (95% HPD)) | | | | | |
|---|---|---|---|---|---|---|---|
| **Parameter** | **Prior** | $p_h$ **= 0.02** | $p_h$ **= 0.05** | $p_h$ **= 0.10** | $p_h$ **= 0.20** | $p_h$ **= 0.50** | $p_h$ **= 0.80** |
| Posterior | | -46359 (-46429 -46296) | -46375 (-46426 -46320) | -46382 (-46434 -46333) | -46383 (-46439 -46335) | -46379 (-46444 -46298) | -46372 (-46434 -46305) |
| Tree height | | 0.394 (0.338 0.465) | 0.375 (0.327 0.432) | 0.371 (0.325 0.430) | 0.363 (0.324 0.427) | 0.362 (0.323 0.425) | 0.369 (0.326 0.431) |
| Clock rate | Uniform(5.0e-4, 2.0e-3) | 7.60e-04 (6.23e-04 8.93e-04) | 7.83e-04 (6.87e-04 8.96e-04) | 7.83e-04 (6.74e-04 9.16e-04) | 8.10e-04 (6.85e-04 9.33e-04) | 7.97e-04 (6.79e-04 9.12e-04) | 7.72e-04 (6.74e-04 8.82e-04) |
| κ | Lognormal(M=1.0, SD=1.25) | 3.741 (3.068 4.507) | 3.739 (3.114 4.505) | 3.708 (3.116 4.532) | 3.704 (3.066 4.392) | 3.733 (3.044 4.530) | 3.765 (3.086 4.450) |
| γ | Exponential(M=1.0) | 3.52e-02 (1.03e-03 8.89e-02) | 3.61e-02 (1.02e-03 8.48e-02) | 3.81e-02 (1.04e-03 8.99e-02) | 3.60e-02 (1.07e-03 9.15e-02) | 3.50e-02 (1.24e-03 8.81e-02) | 3.51e-02 (1.02e-03 8.78e-02) |
| $R_0$ | Lognormal(M=1.5, SD=0.5) | 2.74 (2.47 3.13) | 2.32 (2.11 2.56) | 2.10 (1.90 2.31) | 1.97 (1.80 2.14) | 1.96 (1.71 2.17) | 2.08 (1.81 2.44) |
| α | Uniform(0.0, 2.0) | 0.25 (0.11 0.49) | 0.26 (0.09 0.47) | 0.28 (0.12 0.52) | 0.26 (0.07 0.56) | 0.12 (0.02 0.36) | 0.05 (0.02 0.26) |
| $E_{init}$ | Exponential(M=1.0) | 14.42 (6.51 23.58) | 17.19 (9.51 25.18) | 16.01 (10.05 22.30) | 10.34 (6.67 14.68) | 4.15 (1.98 6.47) | 1.77 (0.37 3.36) |
| $Y_{init}$ | Exponential(M=1.0) | 9.11e-03 (4.69e-03 1.67e-02) | 6.40e-03 (3.83e-03 1.02e-02) | 5.38e-03 (2.87e-03 8.66e-03) | 4.64e-03 (2.82e-03 7.46e-03) | 4.63e-03 (3.01e-03 6.83e-03) | 6.03e-03 (3.66e-03 9.42e-03) |

**Supplementary Table 7: Model parameter priors and estimated values (θ = 1.2)**

| Parameter | Prior | Posterior (Median (95% HPD)) | | | | | |
|---|---|---|---|---|---|---|---|
| | | $p_h$ = 0.02 | $p_h$ = 0.05 | $p_h$ = 0.10 | $p_h$ = 0.20 | $p_h$ = 0.50 | $p_h$ = 0.80 |
| Posterior | | -46351 (-46400 -46310) | -46378 (-46435 -46321) | -46385 (-46436 -46331) | -46355 (-46415 -46297) | -46346 (-46427 -46274) | -46406 (-46456 -46358) |
| Tree height | | 0.422 (0.368 0.488) | 0.407 (0.354 0.474) | 0.392 (0.337 0.456) | 0.379 (0.329 0.437) | 0.367 (0.327 0.422) | 0.381 (0.338 0.437) |
| Clock rate | Uniform(5.0e-4, 2.0e-3) | 7.46e-04 (6.52e-04 8.41e-04) | 7.43e-04 (6.39e-04 8.65e-04) | 7.60e-04 (6.55e-04 8.94e-04) | 7.82e-04 (6.78e-04 9.09e-04) | 7.52e-04 (6.44e-04 8.81e-04) | 6.92e-04 (5.96e-04 8.01e-04) |
| κ | Lognormal(M=1.0, SD=1.25) | 3.686 (3.062 4.395) | 3.683 (3.043 4.458) | 3.713 (3.091 4.422) | 3.717 (3.029 4.462) | 3.689 (3.012 4.392) | 3.669 (3.016 4.399) |
| γ | Exponential(M=1.0) | 3.88e-02 (1.13e-03 8.46e-02) | 3.76e-02 (1.09e-03 8.78e-02) | 3.67e-02 (1.04e-03 9.03e-02) | 3.75e-02 (1.01e-03 9.39e-02) | 3.61e-02 (1.31e-03 8.70e-02) | 3.73e-02 (1.12e-03 9.24e-02) |
| $R_0$ | Lognormal(M=1.5, SD=0.5) | 2.80 (2.51 3.13) | 2.33 (2.11 2.62) | 2.14 (1.91 2.37) | 1.91 (1.69 2.13) | 2.33 (1.75 2.70) | 2.54 (2.11 3.18) |
| α | Uniform(0.0, 2.0) | 0.27 (0.13 0.52) | 0.30 (0.13 0.56) | 0.32 (0.12 0.60) | 0.15 (0.03 0.40) | 0.06 (0.02 0.23) | 0.12 (0.02 0.29) |
| $E_{init}$ | Exponential(M=1.0) | 13.47 (6.82 21.96) | 17.31 (9.80 26.52) | 14.30 (8.12 21.25) | 10.54 (6.14 15.98) | 0.98 (0.28 3.87) | 0.60 (0.04 1.83) |
| $Y_{init}$ | Exponential(M=1.0) | 1.38e-02 (9.24e-03 1.98e-02) | 1.15e-02 (6.26e-03 1.91e-02) | 8.82e-03 (4.80e-03 1.35e-02) | 7.10e-03 (3.68e-03 1.08e-02) | 8.02e-03 (4.16e-03 1.41e-02) | 9.90e-03 (5.60e-03 1.88e-02) |

**Supplementary Table 8: Model parameter priors and estimated values (η = 10)**

| | | Posterior (Median (95% HPD)) | | | | | |
|---|---|---|---|---|---|---|---|
| **Parameter** | **Prior** | $p_h$ **= 0.02** | $p_h$ **= 0.05** | $p_h$ **= 0.10** | $p_h$ **= 0.20** | $p_h$ **= 0.50** | $p_h$ **= 0.80** |
| Posterior | | -46404 (-46444 -46360) | -46403 (-46459 -46341) | -46376 (-46428 -46302) | -46318 (-46398 -46257) | -46355 (-46406 -46301) | -46344 (-46384 -46299) |
| Tree height | | 0.394 (0.344 0.448) | 0.377 (0.330 0.438) | 0.407 (0.357 0.453) | 0.445 (0.387 0.500) | 0.473 (0.419 0.529) | 0.450 (0.403 0.496) |
| Clock rate | Uniform(5.0e-4, 2.0e-3) | 6.75e-04 (5.60e-04 7.81e-04) | 7.29e-04 (5.81e-04 8.75e-04) | 6.62e-04 (5.68e-04 7.53e-04) | 6.16e-04 (5.34e-04 6.91e-04) | 5.46e-04 (5.00e-04 6.20e-04) | 6.31e-04 (5.66e-04 7.05e-04) |
| κ | Lognormal(M=1.0, SD=1.25) | 3.709 (3.074 4.455) | 3.746 (3.119 4.534) | 3.727 (3.068 4.507) | 3.718 (3.045 4.478) | 3.694 (3.139 4.518) | 3.683 (3.090 4.525) |
| γ | Exponential(M=1.0) | 3.56e-02 (1.10e-03 8.78e-02) | 3.37e-02 (1.28e-03 8.77e-02) | 3.76e-02 (1.03e-03 9.27e-02) | 3.76e-02 (1.00e-03 8.98e-02) | 3.46e-02 (1.23e-03 8.77e-02) | 3.57e-02 (1.64e-03 9.15e-02) |
| $R_0$ | Lognormal(M=1.5, SD=0.5, MIN=1.0) | 3.03 (2.64 3.56) | 2.52 (2.16 2.94) | 2.09 (1.88 2.35) | 2.25 (2.09 2.56) | 2.20 (1.96 2.47) | 2.16 (2.02 2.29) |
| α | Uniform(0.0, 2.0) | 0.26 (0.06 0.78) | 0.24 (0.08 0.51) | 0.22 (0.08 0.47) | 0.24 (0.08 0.49) | 0.18 (0.04 0.44) | 0.14 (0.04 0.31) |
| $E_{init}$ | Exponential(M=1.0) | 11.29 (5.93 18.48) | 10.61 (5.33 17.85) | 21.86 (17.25 25.48) | 8.13 (4.73 12.73) | 5.59 (3.58 8.27) | 3.12 (2.83 3.66) |
| $Y_{init}$ | Exponential(M=1.0) | 1.14e-02 (4.64e-03 2.60e-02) | 7.76e-03 (2.49e-03 2.41e-02) | 1.38e-02 (6.91e-03 2.49e-02) | 3.29e-02 (1.32e-02 7.50e-02) | 6.82e-02 (2.18e-02 1.74e-01) | 4.82e-02 (2.75e-02 8.72e-02) |

**Supplementary Table 9: Model parameter priors and estimated values (η = 100)**

| | | Posterior (Median (95% HPD)) | | | | | |
|---|---|---|---|---|---|---|---|
| **Parameter** | **Prior** | $p_h$ **= 0.02** | $p_h$ **= 0.05** | $p_h$ **= 0.10** | $p_h$ **= 0.20** | $p_h$ **= 0.50** | $p_h$ **= 0.80** |
| Posterior | | -46392 (-46447 -46341) | -46339 (-46399 -46293) | -46389 (-46435 -46316) | -46373 (-46436 -46321) | -46379 (-46424 -46334) | -46379 (-46431 -46324) |
| Tree height | | 0.375 (0.329 0.431) | 0.435 (0.338 0.494) | 0.398 (0.344 0.483) | 0.397 (0.343 0.460) | 0.421 (0.362 0.488) | 0.471 (0.410 0.544) |
| Clock rate | Uniform(5.0e-4, 2.0e-3) | 7.06e-04 (5.94e-04 8.61e-04) | 6.03e-04 (5.17e-04 8.14e-04) | 6.20e-04 (5.00e-04 7.27e-04) | 6.33e-04 (5.42e-04 7.35e-04) | 6.04e-04 (5.12e-04 6.88e-04) | 5.38e-04 (5.00e-04 5.97e-04) |
| κ | Lognormal(M=1.0, SD=1.25) | 3.699 (3.055 4.405) | 3.718 (3.009 4.404) | 3.724 (3.077 4.515) | 3.739 (3.098 4.454) | 3.691 (2.991 4.433) | 3.703 (3.061 4.438) |
| γ | Exponential(M=1.0) | 3.50e-02 (1.03e-03 8.47e-02) | 3.59e-02 (1.39e-03 8.92e-02) | 3.53e-02 (1.06e-03 8.63e-02) | 3.72e-02 (1.14e-03 8.99e-02) | 3.52e-02 (1.27e-03 9.11e-02) | 3.39e-02 (1.03e-03 8.23e-02) |
| $R_0$ | Lognormal(M=1.5, SD=0.5, MIN=1.0) | 2.94 (2.57 3.31) | 2.57 (2.29 2.85) | 2.25 (2.00 2.72) | 2.13 (1.95 2.34) | 2.14 (1.95 2.31) | 2.22 (2.07 2.42) |
| α | Uniform(0.0, 2.0) | 0.27 (0.08 0.57) | 0.29 (0.08 0.69) | 0.28 (0.10 0.62) | 0.18 (0.04 0.38) | 0.15 (0.05 0.33) | 0.15 (0.04 0.37) |
| $E_{init}$ | Exponential(M=1.0) | 11.22 (6.25 17.19) | 18.17 (8.53 24.04) | 19.96 (16.05 25.55) | 11.80 (7.88 14.31) | 5.33 (4.11 6.61) | 4.17 (3.75 4.71) |
| $Y_{init}$ | Exponential(M=1.0) | 8.85e-03 (2.72e-03 2.13e-02) | 4.56e-02 (2.61e-03 1.35e-01) | 1.78e-02 (5.16e-03 1.12e-01) | 1.57e-02 (3.12e-03 5.08e-02) | 2.84e-02 (8.84e-03 8.63e-02) | 9.58e-02 (3.68e-02 2.90e-01) |

**Supplementary Table 10: Model parameter priors and estimated values (η = 1000)**

| Parameter | Prior | Posterior (Median (95% HPD)) | | | | | |
|---|---|---|---|---|---|---|---|
| | | **$p_h$ = 0.02** | **$p_h$ = 0.05** | **$p_h$ = 0.10** | **$p_h$ = 0.20** | **$p_h$ = 0.50** | **$p_h$ = 0.80** |
| Posterior | | -46378 (-46419 -46325) | -46356 (-46409 -46309) | -46361 (-46414 -46297) | -46373 (-46435 -46296) | -46405 (-46464 -46317) | -46481 (-46545 -46395) |
| Tree height | | 0.365 (0.336 0.400) | 0.351 (0.325 0.380) | 0.337 (0.323 0.366) | 0.338 (0.323 0.358) | 0.335 (0.322 0.350) | 0.342 (0.322 0.366) |
| Clock rate | Uniform(5.0e-4, 2.0e-3) | 6.80e-04 (5.94e-04 8.04e-04) | 7.55e-04 (6.50e-04 8.52e-04) | 7.89e-04 (6.73e-04 9.25e-04) | 7.82e-04 (6.59e-04 9.04e-04) | 7.63e-04 (6.65e-04 8.81e-04) | 6.63e-04 (5.53e-04 7.97e-04) |
| κ | Lognormal(M=1.0, SD=1.25) | 3.732 (3.088 4.535) | 3.710 (3.079 4.476) | 3.737 (3.064 4.491) | 3.724 (3.049 4.480) | 3.719 (3.081 4.480) | 3.726 (3.053 4.414) |
| γ | Exponential(M=1.0) | 3.53e-02 (1.00e-03 9.50e-02) | 3.64e-02 (1.28e-03 9.05e-02) | 3.56e-02 (1.12e-03 8.55e-02) | 3.40e-02 (1.12e-03 8.97e-02) | 3.71e-02 (1.07e-03 8.64e-02) | 3.57e-02 (1.11e-03 8.62e-02) |
| $R_0$ | Lognormal(M=1.5, SD=0.5, MIN=1.0) | 3.15 (2.77 3.53) | 2.57 (2.32 2.84) | 2.20 (1.98 2.44) | 2.06 (1.84 2.32) | 1.94 (1.70 2.30) | 2.07 (1.79 2.36) |
| α | Uniform(0.0, 2.0) | 0.30 (0.08 0.81) | 0.27 (0.13 0.51) | 0.27 (0.12 0.49) | 0.24 (0.11 0.47) | 0.21 (0.12 0.36) | 0.21 (0.10 0.40) |
| $E_{init}$ | Exponential(M=1.0) | 8.41 (5.17 14.35) | 8.98 (5.18 13.40) | 10.20 (5.42 16.84) | 7.38 (4.43 10.23) | 3.96 (1.25 6.39) | 3.43 (0.78 5.47) |
| $Y_{init}$ | Exponential(M=1.0) | 1.18e-02 (6.39e-03 2.03e-02) | 8.61e-03 (4.53e-03 1.44e-02) | 5.91e-03 (3.89e-03 1.05e-02) | 5.87e-03 (3.52e-03 1.00e-02) | 6.36e-03 (4.12e-03 9.45e-03) | 8.74e-03 (4.48e-03 1.69e-02) |

**Supplementary Table 11: Model parameter priors and estimated values (η = 2500)**

| | | Posterior (Median (95% HPD)) | | | | | |
|---|---|---|---|---|---|---|---|
| **Parameter** | **Prior** | $p_h$ **= 0.02** | $p_h$ **= 0.05** | $p_h$ **= 0.10** | $p_h$ **= 0.20** | $p_h$ **= 0.50** | $p_h$ **= 0.80** |
| Posterior | | -46338 (-46385 -46296) | -46323 (-46371 -46275) | -46335 (-46400 -46275) | -46335 (-46412 -46262) | -46452 (-46525 -46388) | -46448 (-46510 -46390) |
| Tree height | | 0.348 (0.326 0.369) | 0.341 (0.323 0.362) | 0.340 (0.323 0.366) | 0.340 (0.325 0.359) | 0.417 (0.391 0.443) | 0.417 (0.391 0.444) |
| Clock rate | Uniform(5.0e-4, 2.0e-3) | 7.70e-04 (6.52e-04 8.90e-04) | 7.81e-04 (6.84e-04 8.94e-04) | 7.82e-04 (6.62e-04 9.20e-04) | 7.68e-04 (6.39e-04 8.91e-04) | 5.19e-04 (5.00e-04 5.79e-04) | 5.15e-04 (5.00e-04 5.54e-04) |
| κ | Lognormal(M=1.0, SD=1.25) | 3.739 (3.015 4.377) | 3.721 (3.105 4.471) | 3.713 (3.055 4.429) | 3.686 (3.076 4.435) | 3.681 (3.046 4.429) | 3.688 (2.975 4.359) |
| γ | Exponential(M=1.0) | 3.53e-02 (1.17e-03 8.13e-02) | 3.56e-02 (1.05e-03 9.57e-02) | 3.78e-02 (1.01e-03 9.14e-02) | 3.79e-02 (1.23e-03 9.21e-02) | 3.68e-02 (1.02e-03 9.42e-02) | 3.75e-02 (1.08e-03 8.69e-02) |
| $R_0$ | Lognormal(M=1.5, SD=0.5, MIN=1.0) | 2.83 (2.58 3.17) | 2.44 (2.18 2.69) | 2.17 (1.91 2.38) | 2.05 (1.79 2.37) | 1.01 (1.00 1.05) | 1.06 (1.00 1.14) |
| α | Uniform(0.0, 2.0) | 0.27 (0.11 0.54) | 0.27 (0.14 0.48) | 0.30 (0.15 0.50) | 0.29 (0.17 0.44) | 0.55 (0.38 0.74) | 0.41 (0.25 0.59) |
| $E_{init}$ | Exponential(M=1.0) | 10.88 (6.51 15.44) | 10.56 (6.93 15.36) | 9.31 (4.64 13.16) | 4.80 (2.38 8.79) | 2.01 (1.40 2.63) | 1.82 (1.27 2.39) |
| $Y_{init}$ | Exponential(M=1.0) | 1.46e-02 (8.50e-03 2.38e-02) | 1.10e-02 (6.63e-03 1.60e-02) | 1.01e-02 (5.00e-03 1.75e-02) | 9.90e-03 (5.06e-03 1.87e-02) | 8.67e-02 (4.53e-02 1.38e-01) | 9.12e-02 (5.60e-02 1.36e-01) |

**Supplementary Table 12: Model parameter priors and estimated values (η = 5000)**

| | | Posterior (Median (95% HPD)) | | | | | |
|---|---|---|---|---|---|---|---|
| **Parameter** | **Prior** | $p_h$ **= 0.02** | $p_h$ **= 0.05** | $p_h$ **= 0.10** | $p_h$ **= 0.20** | $p_h$ **= 0.50** | $p_h$ **= 0.80** |
| Posterior | | -46335 (-46377 -46289) | -46327 (-46379 -46279) | -46326 (-46382 -46263) | -46306 (-46363 -46253) | -46365 (-46424 -46315) | -46428 (-46478 -46380) |
| Tree height | | 0.355 (0.327 0.381) | 0.348 (0.328 0.370) | 0.344 (0.326 0.364) | 0.444 (0.425 0.466) | 0.436 (0.412 0.464) | 0.439 (0.414 0.463) |
| Clock rate | Uniform(5.0e-4, 2.0e-3) | 7.21e-04 (6.21e-04 8.30e-04) | 7.66e-04 (6.57e-04 8.80e-04) | 7.87e-04 (6.71e-04 9.08e-04) | 5.07e-04 (5.00e-04 5.30e-04) | 5.07e-04 (5.00e-04 5.29e-04) | 5.05e-04 (5.00e-04 5.22e-04) |
| κ | Lognormal(M=1.0, SD=1.25) | 3.712 (3.017 4.416) | 3.726 (3.050 4.425) | 3.725 (3.073 4.472) | 3.696 (3.057 4.427) | 3.682 (3.055 4.400) | 3.700 (3.058 4.495) |
| γ | Exponential(M=1.0) | 3.96e-02 (1.12e-03 9.39e-02) | 3.38e-02 (1.15e-03 8.39e-02) | 3.49e-02 (1.26e-03 8.90e-02) | 3.69e-02 (1.16e-03 9.11e-02) | 3.67e-02 (1.02e-03 8.53e-02) | 3.47e-02 (1.01e-03 8.95e-02) |
| $R_0$ | Lognormal(M=1.5, SD=0.5, MIN=1.0) | 2.86 (2.58 3.19) | 2.39 (2.14 2.68) | 2.25 (1.95 2.52) | 1.02 (1.00 1.06) | 1.03 (1.00 1.09) | 1.11 (1.03 1.18) |
| α | Uniform(0.0, 2.0) | 0.27 (0.11 0.52) | 0.32 (0.16 0.54) | 0.32 (0.19 0.54) | 0.82 (0.62 0.99) | 0.68 (0.49 0.88) | 0.49 (0.34 0.68) |
| $E_{init}$ | Exponential(M=1.0) | 11.61 (6.50 17.27) | 10.88 (4.52 17.09) | 5.95 (2.84 9.63) | 2.55 (2.12 2.82) | 2.04 (1.51 2.60) | 1.57 (1.14 2.10) |
| $Y_{init}$ | Exponential(M=1.0) | 2.22e-02 (1.37e-02 3.43e-02) | 1.79e-02 (9.95e-03 2.86e-02) | 1.27e-02 (8.02e-03 1.83e-02) | 1.65e-01 (1.09e-01 2.48e-01) | 1.49e-01 (9.60e-02 2.29e-01) | 1.49e-01 (9.54e-02 2.21e-01) |

**Supplementary Table 13. Sequencing primer list**

| name | seq | length |
|---|---|---|
| nCoV-2019_1_LEFT | ACCAACCAACTTTCGATCTCTTGT | 24 |
| nCoV-2019_1_RIGHT | CATCTTTAAGATGTTGACGTGCCTC | 25 |
| nCoV-2019_2_LEFT | CTGTTTTACAGGTTCGCGACGT | 22 |
| nCoV-2019_2_RIGHT | TAAGGATCAGTGCCAAGCTCGT | 22 |
| nCoV-2019_3_LEFT | CGGTAATAAAGGAGCTGGTGGC | 22 |
| nCoV-2019_3_RIGHT | AAGGTGTCTGCAATTCATAGCTCT | 24 |
| nCoV-2019_4_LEFT | GGTGTATACTGCTGCCGTGAAC | 22 |
| nCoV-2019_4_RIGHT | CACAAGTAGTGGCACCTTCTTTAGT | 25 |
| nCoV-2019_5_LEFT | TGGTGAAACTTCATGGCAGACG | 22 |
| nCoV-2019_5_RIGHT | ATTGATGTTGACTTTCTCTTTTTGGAGT | 28 |
| nCoV-2019_6_LEFT | GGTGTTGTTGGAGAAGGTTCCG | 22 |
| nCoV-2019_6_RIGHT | TAGCGGCCTTCTGTAAAACACG | 22 |
| nCoV-2019_7_LEFT | ATCAGAGGCTGCTCGTGTTGTA | 22 |
| nCoV-2019_7_LEFT_alt0 | CATTTGCATCAGAGGCTGCTCG | 22 |
| nCoV-2019_7_RIGHT | TGCACAGGTGACAATTTGTCCA | 22 |
| nCoV-2019_7_RIGHT_alt5 | AGGTGACAATTTGTCCACCGAC | 22 |
| nCoV-2019_8_LEFT | AGAGTTTCTTAGAGACGGTTGGGA | 24 |
| nCoV-2019_8_RIGHT | GCTTCAACAGCTTCACTAGTAGGT | 24 |
| nCoV-2019_9_LEFT | TCCCACAGAAGTGTTAACAGAGGA | 24 |
| nCoV-2019_9_LEFT_alt4 | TTCCCACAGAAGTGTTAACAGAGG | 24 |
| nCoV-2019_9_RIGHT | ATGACAGCATCTGCCACAACAC | 22 |
| nCoV-2019_9_RIGHT_alt2 | GACAGCATCTGCCACAACACAG | 22 |
| nCoV-2019_10_LEFT | TGAGAAGTGCTCTGCCTATACAGT | 24 |
| nCoV-2019_10_RIGHT | TCATCTAACCAATCTTCTTCTTGCTCT | 27 |
| nCoV-2019_11_LEFT | GGAATTTGGTGCCACTTCTGCT | 22 |
| nCoV-2019_11_RIGHT | TCATCAGATTCAACTTGCATGGCA | 24 |
| nCoV-2019_12_LEFT | AAACATGGAGGAGGTGTTGCAG | 22 |
| nCoV-2019_12_RIGHT | TTCACTCTTCATTTCCAAAAAGCTTGA | 27 |
| nCoV-2019_13_LEFT | TCGCACAAATGTCTACTTAGCTGT | 24 |
| nCoV-2019_13_RIGHT | ACCACAGCAGTTAAAACACCCT | 22 |
| nCoV-2019_14_LEFT | CATCCAGATTCTGCCACTCTTGT | 23 |
| nCoV-2019_14_LEFT_alt4 | TGGCAATCTTCATCCAGATTCTGC | 24 |
| nCoV-2019_14_RIGHT | AGTTTCCACACAGACAGGCATT | 22 |
| nCoV-2019_14_RIGHT_alt2 | TGCGTGTTTCTTCTGCATGTGC | 22 |
| nCoV-2019_15_LEFT | ACAGTGCTTAAAAAGTGTAAAAGTGCC | 27 |
| nCoV-2019_15_LEFT_alt1 | AGTGCTTAAAAAGTGTAAAAGTGCCT | 26 |
| nCoV-2019_15_RIGHT | AACAGAAACTGTAGCTGGCACT | 22 |
| nCoV-2019_15_RIGHT_alt3 | ACTGTAGCTGGCACTTTGAGAGA | 23 |
| nCoV-2019_16_LEFT | AATTTGGAAGAAGCTGCTCGGT | 22 |
| nCoV-2019_16_RIGHT | CACAACTTGCGTGTGGAGGTTA | 22 |
| nCoV-2019_17_LEFT | CTTCTTTCTTTGAGAGAAGTGAGGACT | 27 |
| nCoV-2019_17_RIGHT | TTTGTTGGAGTGTTAACAATGCAGT | 25 |
| nCoV-2019_18_LEFT | TGGAAATACCCACAAGTTAATGGTTTAAC | 29 |
| nCoV-2019_18_LEFT_alt2 | ACTTCTATTAAATGGGCAGATAACAACTGT | 30 |
| nCoV-2019_18_RIGHT | AGCTTGTTTACCACACGTACAAGG | 24 |

| | | |
|---|---|---|
| nCoV-2019_18_RIGHT_alt1 | GCTTGTTTACCACACGTACAAGG | 23 |
| nCoV-2019_19_LEFT | GCTGTTATGTACATGGGCACACT | 23 |
| nCoV-2019_19_RIGHT | TGTCCAACTTAGGGTCAATTTCTGT | 25 |
| nCoV-2019_20_LEFT | ACAAAGAAAACAGTTACACAACAACCA | 27 |
| nCoV-2019_20_RIGHT | ACGTGGCTTTATTAGTTGCATTGTT | 25 |
| nCoV-2019_21_LEFT | TGGCTATTGATTATAAACACTACACACCC | 29 |
| nCoV-2019_21_LEFT_alt2 | GGCTATTGATTATAAACACTACACACCCT | 29 |
| nCoV-2019_21_RIGHT | TAGATCTGTGTGGCCAACCTCT | 22 |
| nCoV-2019_21_RIGHT_alt0 | GATCTGTGTGGCCAACCTCTTC | 22 |
| nCoV-2019_22_LEFT | ACTACCGAAGTTGTAGGAGACATTATACT | 29 |
| nCoV-2019_22_RIGHT | ACAGTATTCTTTGCTATAGTAGTCGGC | 27 |
| nCoV-2019_23_LEFT | ACAACTACTAACATAGTTACACGGTGT | 27 |
| nCoV-2019_23_RIGHT | ACCAGTACAGTAGGTTGCAATAGTG | 25 |
| nCoV-2019_24_LEFT | AGGCATGCCTTCTTACTGTACTG | 23 |
| nCoV-2019_24_RIGHT | ACATTCTAACCATAGCTGAAATCGGG | 26 |
| nCoV-2019_25_LEFT | GCAATTGTTTTTCAGCTATTTTGCAGT | 27 |
| nCoV-2019_25_RIGHT | ACTGTAGTGACAAGTCTCTCGCA | 23 |
| nCoV-2019_26_LEFT | TTGTGATACATTCTGTGCTGGTAGT | 25 |
| nCoV-2019_26_RIGHT | TCCGCACTATCACCAACATCAG | 22 |
| nCoV-2019_27_LEFT | ACTACAGTCAGCTTATGTGTCAACC | 25 |
| nCoV-2019_27_RIGHT | AATACAAGCACCAAGGTCACGG | 22 |
| nCoV-2019_28_LEFT | ACATAGAAGTTACTGGCGATAGTTGT | 26 |
| nCoV-2019_28_RIGHT | TGTTTAGACATGACATGAACAGGTGT | 26 |
| nCoV-2019_29_LEFT | ACTTGTGTTCCTTTTTGTTGCTGC | 24 |
| nCoV-2019_29_RIGHT | AGTGTACTCTATAAGTTTTGATGGTGTGT | 29 |
| nCoV-2019_30_LEFT | GCACAACTAATGGTGACTTTTTGCA | 25 |
| nCoV-2019_30_RIGHT | ACCACTAGTAGATACACAAACACCAG | 26 |
| nCoV-2019_31_LEFT | TTCTGAGTACTGTAGGCACGGC | 22 |
| nCoV-2019_31_RIGHT | ACAGAATAAACACCAGGTAAGAATGAGT | 28 |
| nCoV-2019_32_LEFT | TGGTGAATACAGTCATGTAGTTGCC | 25 |
| nCoV-2019_32_RIGHT | AGCACATCACTACGCAACTTTAGA | 24 |
| nCoV-2019_33_LEFT | ACTTTTGAAGAAGCTGCGCTGT | 22 |
| nCoV-2019_33_RIGHT | TGGACAGTAAACTACGTCATCAAGC | 25 |
| nCoV-2019_34_LEFT | TCCCATCTGGTAAAGTTGAGGGT | 23 |
| nCoV-2019_34_RIGHT | AGTGAAATTGGGCCTCATAGCA | 22 |
| nCoV-2019_35_LEFT | TGTTCGCATTCAACCAGGACAG | 22 |
| nCoV-2019_35_RIGHT | ACTTCATAGCCACAAGGTTAAAGTCA | 26 |
| nCoV-2019_36_LEFT | TTAGCTTGGTTGTACGCTGCTG | 22 |
| nCoV-2019_36_RIGHT | GAACAAAGACCATTGAGTACTCTGGA | 26 |
| nCoV-2019_37_LEFT | ACACACCACTGGTTGTTACTCAC | 23 |
| nCoV-2019_37_RIGHT | GTCCACACTCTCCTAGCACCAT | 22 |
| nCoV-2019_38_LEFT | ACTGTGTTATGTATGCATCAGCTGT | 25 |
| nCoV-2019_38_RIGHT | CACCAAGAGTCAGTCTAAAGTAGCG | 25 |
| nCoV-2019_39_LEFT | AGTATTGCCCTATTTTCTTCATAACTGGT | 29 |
| nCoV-2019_39_RIGHT | TGTAACTGGACACATTGAGCCC | 22 |
| nCoV-2019_40_LEFT | TGCACATCAGTAGTCTTACTCTCAGT | 26 |
| nCoV-2019_40_RIGHT | CATGGCTGCATCACGGTCAAAT | 22 |
| nCoV-2019_41_LEFT | GTTCCCTTCCATCATATGCAGCT | 23 |

| | | |
|---|---|---|
| nCoV-2019_41_RIGHT | TGGTATGACAACCATTAGTTTGGCT | 25 |
| nCoV-2019_42_LEFT | TGCAAGAGATGGTTGTGTTCCC | 22 |
| nCoV-2019_42_RIGHT | CCTACCTCCCTTTGTTGTGTTGT | 23 |
| nCoV-2019_43_LEFT | TACGACAGATGTCTTGTGCTGC | 22 |
| nCoV-2019_43_RIGHT | AGCAGCATCTACAGCAAAAGCA | 22 |
| nCoV-2019_44_LEFT | TGCCACAGTACGTCTACAAGCT | 22 |
| nCoV-2019_44_LEFT_alt3 | CCACAGTACGTCTACAAGCTGG | 22 |
| nCoV-2019_44_RIGHT | AACCTTTCCACATACCGCAGAC | 22 |
| nCoV-2019_44_RIGHT_alt0 | CGCAGACGGTACAGACTGTGTT | 22 |
| nCoV-2019_45_LEFT | TACCTACAACTTGTGCTAATGACCC | 25 |
| nCoV-2019_45_LEFT_alt2 | AGTATGTACAAATACCTACAACTTGTGCT | 29 |
| nCoV-2019_45_RIGHT | AAATTGTTTCTTCATGTTGGTAGTTAGAGA | 30 |
| nCoV-2019_45_RIGHT_alt7 | TTCATGTTGGTAGTTAGAGAAAGTGTGTC | 29 |
| nCoV-2019_46_LEFT | TGTCGCTTCCAAGAAAAGGACG | 22 |
| nCoV-2019_46_LEFT_alt1 | CGCTTCCAAGAAAAGGACGAAGA | 23 |
| nCoV-2019_46_RIGHT | CACGTTCACCTAAGTTGGCGTA | 22 |
| nCoV-2019_46_RIGHT_alt2 | CACGTTCACCTAAGTTGGCGTAT | 23 |
| nCoV-2019_47_LEFT | AGGACTGGTATGATTTTGTAGAAAACCC | 28 |
| nCoV-2019_47_RIGHT | AATAACGGTCAAAGAGTTTTAACCTCTC | 28 |
| nCoV-2019_48_LEFT | TGTTGACACTGACTTAACAAAGCCT | 25 |
| nCoV-2019_48_RIGHT | TAGATTACCAGAAGCAGCGTGC | 22 |
| nCoV-2019_49_LEFT | AGGAATTACTTGTGTATGCTGCTGA | 25 |
| nCoV-2019_49_RIGHT | TGACGATGACTTGGTTAGCATTAATACA | 28 |
| nCoV-2019_50_LEFT | GTTGATAAGTACTTTGATTGTTACGATGGT | 30 |
| nCoV-2019_50_RIGHT | TAACATGTTGTGCCAACCACCA | 22 |
| nCoV-2019_51_LEFT | TCAATAGCCGCCACTAGAGGAG | 22 |
| nCoV-2019_51_RIGHT | AGTGCATTAACATTGGCCGTGA | 22 |
| nCoV-2019_52_LEFT | CATCAGGAGATGCCACAACTGC | 22 |
| nCoV-2019_52_RIGHT | GTTGAGAGCAAAATTCATGAGGTCC | 25 |
| nCoV-2019_53_LEFT | AGCAAAATGTTGGACTGAGACTGA | 24 |
| nCoV-2019_53_RIGHT | AGCCTCATAAAACTCAGGTTCCC | 23 |
| nCoV-2019_54_LEFT | TGAGTTAACAGGACACATGTTAGACA | 26 |
| nCoV-2019_54_RIGHT | AACCAAAAACTTGTCCATTAGCACA | 25 |
| nCoV-2019_55_LEFT | ACTCAACTTTACTTAGGAGGTATGAGCT | 28 |
| nCoV-2019_55_RIGHT | GGTGTACTCTCCTATTTGTACTTTACTGT | 29 |
| nCoV-2019_56_LEFT | ACCTAGACCACCACTTAACCGA | 22 |
| nCoV-2019_56_RIGHT | ACACTATGCGAGCAGAAGGGTA | 22 |
| nCoV-2019_57_LEFT | ATTCTACACTCCAGGGACCACC | 22 |
| nCoV-2019_57_RIGHT | GTAATTGAGCAGGGTCGCCAAT | 22 |
| nCoV-2019_58_LEFT | TGATTTGAGTGTTGTCAATGCCAGA | 25 |
| nCoV-2019_58_RIGHT | CTTTTCTCCAAGCAGGGTTACGT | 23 |
| nCoV-2019_59_LEFT | TCACGCATGATGTTTCATCTGCA | 23 |
| nCoV-2019_59_RIGHT | AAGAGTCCTGTTACATTTTCAGCTTG | 26 |
| nCoV-2019_60_LEFT | TGATAGAGACCTTTATGACAAGTTGCA | 27 |
| nCoV-2019_60_RIGHT | GGTACCAACAGCTTCTCTAGTAGC | 24 |
| nCoV-2019_61_LEFT | TGTTTATCACCCGCGAAGAAGC | 22 |
| nCoV-2019_61_RIGHT | ATCACATAGACAACAGGTGCGC | 22 |
| nCoV-2019_62_LEFT | GGCACATGGCTTTGAGTTGACA | 22 |

| | | |
|---|---|---|
| nCoV-2019_62_RIGHT | GTTGAACCTTTCTACAAGCCGC | 22 |
| nCoV-2019_63_LEFT | TGTTAAGCGTGTTGACTGGACT | 22 |
| nCoV-2019_63_RIGHT | ACAAACTGCCACCATCACAACC | 22 |
| nCoV-2019_64_LEFT | TCGATAGATATCCTGCTAATTCCATTGT | 28 |
| nCoV-2019_64_RIGHT | AGTCTTGTAAAAGTGTTCCAGAGGT | 25 |
| nCoV-2019_65_LEFT | GCTGGCTTTAGCTTGTGGGTTT | 22 |
| nCoV-2019_65_RIGHT | TGTCAGTCATAGAACAAACACCAATAGT | 28 |
| nCoV-2019_66_LEFT | GGGTGTGGACATTGCTGCTAAT | 22 |
| nCoV-2019_66_RIGHT | TCAATTTCCATTTGACTCCTGGGT | 24 |
| nCoV-2019_67_LEFT | GTTGTCCAACAATTACCTGAAACTTACT | 28 |
| nCoV-2019_67_RIGHT | CAACCTTAGAAACTACAGATAAATCTTGGG | 30 |
| nCoV-2019_68_LEFT | ACAGGTTCATCTAAGTGTGTGTGT | 24 |
| nCoV-2019_68_RIGHT | CTCCTTTATCAGAACCAGCACCA | 23 |
| nCoV-2019_69_LEFT | TGTCGCAAAATATACTCAACTGTGTCA | 27 |
| nCoV-2019_69_RIGHT | TCTTTATAGCCACGGAACCTCCA | 23 |
| nCoV-2019_70_LEFT | ACAAAAGAAAATGACTCTAAAGAGGGTTT | 29 |
| nCoV-2019_70_RIGHT | TGACCTTCTTTTAAAGACATAACAGCAG | 28 |
| nCoV-2019_71_LEFT | ACAAATCCAATTCAGTTGTCTTCCTATTC | 29 |
| nCoV-2019_71_RIGHT | TGGAAAAGAAAGGTAAGAACAAGTCCT | 27 |
| nCoV-2019_72_LEFT | ACACGTGGTGTTTATTACCCTGAC | 24 |
| nCoV-2019_72_RIGHT | ACTCTGAACTCACTTTCCATCCAAC | 25 |
| nCoV-2019_73_LEFT | CAATTTTGTAATGATCCATTTTTGGGTGT | 29 |
| nCoV-2019_73_RIGHT | CACCAGCTGTCCAACCTGAAGA | 22 |
| nCoV-2019_74_LEFT | ACATCACTAGGTTTCAAACTTTACTTGC | 28 |
| nCoV-2019_74_RIGHT | GCAACACAGTTGCTGATTCTCTTC | 24 |
| nCoV-2019_75_LEFT | AGAGTCCAACCAACAGAATCTATTGT | 26 |
| nCoV-2019_75_RIGHT | ACCACCAACCTTAGAATCAAGATTGT | 26 |
| nCoV-2019_76_LEFT | AGGGCAAACTGGAAAGATTGCT | 22 |
| nCoV-2019_76_LEFT_alt3 | GGGCAAACTGGAAAGATTGCTGA | 23 |
| nCoV-2019_76_RIGHT | ACACCTGTGCCTGTTAAACCAT | 22 |
| nCoV-2019_76_RIGHT_alt0 | ACCTGTGCCTGTTAAACCATTGA | 23 |
| nCoV-2019_77_LEFT | CCAGCAACTGTTTGTGGACCTA | 22 |
| nCoV-2019_77_RIGHT | CAGCCCCTATTAAACAGCCTGC | 22 |
| nCoV-2019_78_LEFT | CAACTTACTCCTACTTGGCGTGT | 23 |
| nCoV-2019_78_RIGHT | TGTGTACAAAAACTGCCATATTGCA | 25 |
| nCoV-2019_79_LEFT | GTGGTGATTCAACTGAATGCAGC | 23 |
| nCoV-2019_79_RIGHT | CATTTCATCTGTGAGCAAAGGTGG | 24 |
| nCoV-2019_80_LEFT | TTGCCTTGGTGATATTGCTGCT | 22 |
| nCoV-2019_80_RIGHT | TGGAGCTAAGTTGTTTAACAAGCG | 24 |
| nCoV-2019_81_LEFT | GCACTTGGAAAACTTCAAGATGTGG | 25 |
| nCoV-2019_81_RIGHT | GTGAAGTTCTTTTCTTGTGCAGGG | 24 |
| nCoV-2019_82_LEFT | GGGCTATCATCTTATGTCCTTCCCT | 25 |
| nCoV-2019_82_RIGHT | TGCCAGAGATGTCACCTAAATCAA | 24 |
| nCoV-2019_83_LEFT | TCCTTTGCAACCTGAATTAGACTCA | 25 |
| nCoV-2019_83_RIGHT | TTTGACTCCTTTGAGCACTGGC | 22 |
| nCoV-2019_84_LEFT | TGCTGTAGTTGTCTCAAGGGCT | 22 |
| nCoV-2019_84_RIGHT | AGGTGTGAGTAAACTGTTACAAACAAC | 27 |
| nCoV-2019_85_LEFT | ACTAGCACTCTCCAAGGGTGTT | 22 |

| | | |
|---|---|---|
| nCoV-2019_85_RIGHT | ACACAGTCTTTTACTCCAGATTCCC | 25 |
| nCoV-2019_86_LEFT | TCAGGTGATGGCACAACAAGTC | 22 |
| nCoV-2019_86_RIGHT | ACGAAAGCAAGAAAAAGAAGTACGC | 25 |
| nCoV-2019_87_LEFT | CGACTACTAGCGTGCCTTTGTA | 22 |
| nCoV-2019_87_RIGHT | ACTAGGTTCCATTGTTCAAGGAGC | 24 |
| nCoV-2019_88_LEFT | CCATGGCAGATTCCAACGGTAC | 22 |
| nCoV-2019_88_RIGHT | TGGTCAGAATAGTGCCATGGAGT | 23 |
| nCoV-2019_89_LEFT | GTACGCGTTCCATGTGGTCATT | 22 |
| nCoV-2019_89_LEFT_alt2 | CGCGTTCCATGTGGTCATTCAA | 22 |
| nCoV-2019_89_RIGHT | ACCTGAAAGTCAACGAGATGAAACA | 25 |
| nCoV-2019_89_RIGHT_alt4 | ACGAGATGAAACATCTGTTGTCACT | 25 |
| nCoV-2019_90_LEFT | ACACAGACCATTCCAGTAGCAGT | 23 |
| nCoV-2019_90_RIGHT | TGAAATGGTGAATTGCCCTCGT | 22 |
| nCoV-2019_91_LEFT | TCACTACCAAGAGTGTGTTAGAGGT | 25 |
| nCoV-2019_91_RIGHT | TTCAAGTGAGAACCAAAAGATAATAAGCA | 29 |
| nCoV-2019_92_LEFT | TTTGTGCTTTTTAGCCTTTCTGCT | 24 |
| nCoV-2019_92_RIGHT | AGGTTCCTGGCAATTAATTGTAAAAGG | 27 |
| nCoV-2019_93_LEFT | TGAGGCTGGTTCTAAATCACCCA | 23 |
| nCoV-2019_93_RIGHT | AGGTCTTCCTTGCCATGTTGAG | 22 |
| nCoV-2019_94_LEFT | GGCCCCAAGGTTTACCCAATAA | 22 |
| nCoV-2019_94_RIGHT | TTTGGCAATGTTGTTCCTTGAGG | 23 |
| nCoV-2019_95_LEFT | TGAGGGAGCCTTGAATACACCA | 22 |
| nCoV-2019_95_RIGHT | CAGTACGTTTTTGCCGAGGCTT | 22 |
| nCoV-2019_96_LEFT | GCCAACAACAACAAGGCCAAAC | 22 |
| nCoV-2019_96_RIGHT | TAGGCTCTGTTGGTGGGAATGT | 22 |
| nCoV-2019_97_LEFT | TGGATGACAAAGATCCAAATTTCAAAGA | 28 |
| nCoV-2019_97_RIGHT | ACACACTGATTAAAGATTGCTATGTGAG | 28 |
| nCoV-2019_98_LEFT | AACAATTGCAACAATCCATGAGCA | 24 |
| nCoV-2019_98_RIGHT | TTCTCCTAAGAAGCTATTAAAATCACATGG | 30 |
