# Supplementary material for: Full genome viral sequences inform patterns of SARS-CoV-2 spread into and within Israel
Source: Nat Commun. 2020 Nov 2;11:5518. doi: 10.1038/s41467-020-19248-0 (PMC7606475; doi:10.1038/s41467-020-19248-0)
Supplement: Supplementary file 3 — Reporting Summary [file 41467_2020_19248_MOESM3_ESM.pdf]

nature research

Corresponding author(s): Adi Stern

Last updated by author(s): Sep 21, 2020

Reporting Summary

Nature Research wishes to improve the reproducibility of the work that we publish. This form provides structure for consistency and transparency in reporting. For further information on Nature Research policies, see our Editorial Policies and the Editorial Policy Checklist.

Statistics

For all statistical analyses, confirm that the following items are present in the figure legend, table legend, main text, or Methods section.

n/a	Confirmed	
☐	☒	The exact sample size (*n*) for each experimental group/condition, given as a discrete number and unit of measurement
☐	☒	A statement on whether measurements were taken from distinct samples or whether the same sample was measured repeatedly
☐	☒	The statistical test(s) used AND whether they are one- or two-sided<br>*Only common tests should be described solely by name; describe more complex techniques in the Methods section.*
☐	☒	A description of all covariates tested
☐	☒	A description of any assumptions or corrections, such as tests of normality and adjustment for multiple comparisons
☐	☒	A full description of the statistical parameters including central tendency (e.g. means) or other basic estimates (e.g. regression coefficient) AND variation (e.g. standard deviation) or associated estimates of uncertainty (e.g. confidence intervals)
☐	☒	For null hypothesis testing, the test statistic (e.g. *F*, *t*, *r*) with confidence intervals, effect sizes, degrees of freedom and *P* value noted<br>*Give P values as exact values whenever suitable.*
☐	☒	For Bayesian analysis, information on the choice of priors and Markov chain Monte Carlo settings
☐	☒	For hierarchical and complex designs, identification of the appropriate level for tests and full reporting of outcomes
☒	☐	Estimates of effect sizes (e.g. Cohen's *d*, Pearson's *r*), indicating how they were calculated

Our web collection on statistics for biologists contains articles on many of the points above.

Software and code

Policy information about availability of computer code

Data collection	None
Data analysis	pTrimmer v1.3.1, AccuNGS, NextStrain ncov pipeline (latest build as for May 2020),IQTree under Augur version v10.0.2, BEAST2 v2.6.2, PhyDyn v1.3.6, Python v3.7.4, BEAUti v2.6.3, CoupledMCMC v1.0.2, TreeAnnotator v2.6.3, Matplotlib v3.2.1, Seaborn v0.10.1,Baltic v0.1.0, ggtree v2.0.4 and customized scripts that are all available at https://github.com/SternLabTAU/SARSCOV2NGS. All software used is cited.

For manuscripts utilizing custom algorithms or software that are central to the research but not yet described in published literature, software must be made available to editors and reviewers. We strongly encourage code deposition in a community repository (e.g. GitHub). See the Nature Research guidelines for submitting code & software for further information.

Data

Policy information about availability of data

All manuscripts must include a data availability statement. This statement should provide the following information, where applicable:

- Accession codes, unique identifiers, or web links for publicly available datasets
- A list of figures that have associated raw data
- A description of any restrictions on data availability

Data that support the findings of this study have been deposited in the relevant databases: a viral sequence per each patient sample was deposited in the GISAID database (https://www.gisaid.org) with accession numbers EPI_ISL_447258 - EPI_ISL_447469. The raw sequencing reads were deposited in the NCBI Sequence Read Archive (SRA) database under BioProject accession number PRJNA647529 (https://www.ncbi.nlm.nih.gov/bioproject/?term=PRJNA647529). The reference genome of SARS-CoV-2, ID MN908947, was downloaded from GenBank (https://www.ncbi.nlm.nih.gov/genbank/). A list of all sequence accession numbers used in this study, Beast XML configurations and outputs are available at: https://github.com/SternLabTAU/SARSCOV2NGS. Source data used to generate all figures are provided with this paper.

Field-specific reporting

Please select the one below that is the best fit for your research. If you are not sure, read the appropriate sections before making your selection.

☐ Life sciences ☐ Behavioural & social sciences ☒ Ecological, evolutionary & environmental sciences

For a reference copy of the document with all sections, see nature.com/documents/nr-reporting-summary-flat.pdf

Ecological, evolutionary & environmental sciences study design

All studies must disclose on these points even when the disclosure is negative.

Study description	Samples of SARS-CoV2 infected individuals were collected and virus genomes were sequenced
Research sample	RNA extracted from nasopharynx swabs of SARS-CoV2 positive individuals was collected randomly by medical centers in Israel, excluding relatives and spanning different dates across the time interval of March through April. The data included both males and females in the same ratio aged 0 to 99. This sampling procedure was done in order to obtain independent, representative sample of the population of Israel needed for later model assumptions.
Sampling strategy	We aimed at reaching a sample size of 200, with more or less equal numbers of samples from each of the collaborating medical centers. The number of sequences is approximately 1.5% of the total number of reported cases on April 22 which was satisfying for our statistical approach.
Data collection	Extracted RNA was collected directly from Israel's medical centers to sequencing in the Genomic unit at the Technion. Metadata of samples was provided by medical centers and included age, sex, and geographical district.
Timing and spatial scale	Samples were collected starting on March 17, 2020 till April 22, 2020 from six medical centers spanning the entire geography of Israel. Samples dates were collected based on data availability in medical centers and were selected to uniformly (as possible) cover the specified time interval.
Data exclusions	Six samples were excluded from the phylodynamics analysis: samples suspected to be from the same household, samples with consecutive identifiers, or identical samples with similar identifiers and similar dates. Only one sample from a given household was chosen randomly.
Reproducibility	There was no need to repeat the experiment (i.e., sequencing).
Randomization	No allocation to groups was performed in the study.
Blinding	All samples were anonymized, and were assigned a random identifier by the medical centers.

Did the study involve field work? ☐ Yes ☒ No

Reporting for specific materials, systems and methods

We require information from authors about some types of materials, experimental systems and methods used in many studies. Here, indicate whether each material, system or method listed is relevant to your study. If you are not sure if a list item applies to your research, read the appropriate section before selecting a response.

Materials & experimental systems

n/a	Involved in the study	
☒	☐	Antibodies
☒	☐	Eukaryotic cell lines
☒	☐	Palaeontology and archaeology
☒	☐	Animals and other organisms
☐	☒	Human research participants
☒	☐	Clinical data
☒	☐	Dual use research of concern

Methods

n/a	Involved in the study	
☒	☐	ChIP-seq
☒	☐	Flow cytometry
☒	☐	MRI-based neuroimaging

Human research participants

Policy information about studies involving human research participants

Population characteristics	All samples were collected randomly from the entire geography of Israel, including both male and female from ages between 0 to 99. No other clinical information was provided on these samples.
Recruitment	Samples were chosen randomly from existing positive samples
Ethics oversight	An exemption from institutional review board approval was determined by the Israeli Ministry of Health as part of an active epidemiological investigation, based on use of retrospective anonymous data only and no medical intervention. This included exemption from informed consent. The study was further approved by the Tel-Aviv University ethics committee (approval 0001274-1).

Note that full information on the approval of the study protocol must also be provided in the manuscript.
